# Impaired complex I repair causes recessive Leber’s hereditary optic neuropathy

**DOI:** 10.1172/JCI138267

**Published:** 2021-03-15

**Authors:** Sarah L. Stenton, Natalia L. Sheremet, Claudia B. Catarino, Natalia A. Andreeva, Zahra Assouline, Piero Barboni, Ortal Barel, Riccardo Berutti, Igor Bychkov, Leonardo Caporali, Mariantonietta Capristo, Michele Carbonelli, Maria L. Cascavilla, Peter Charbel Issa, Peter Freisinger, Sylvie Gerber, Daniele Ghezzi, Elisabeth Graf, Juliana Heidler, Maja Hempel, Elise Heon, Yulya S. Itkis, Elisheva Javasky, Josseline Kaplan, Robert Kopajtich, Cornelia Kornblum, Reka Kovacs-Nagy, Tatiana D. Krylova, Wolfram S. Kunz, Chiara La Morgia, Costanza Lamperti, Christina Ludwig, Pedro F. Malacarne, Alessandra Maresca, Johannes A. Mayr, Jana Meisterknecht, Tatiana A. Nevinitsyna, Flavia Palombo, Ben Pode-Shakked, Maria S. Shmelkova, Tim M. Strom, Francesca Tagliavini, Michal Tzadok, Amelie T. van der Ven, Catherine Vignal-Clermont, Matias Wagner, Ekaterina Y. Zakharova, Nino V. Zhorzholadze, Jean-Michel Rozet, Valerio Carelli, Polina G. Tsygankova, Thomas Klopstock, Ilka Wittig, Holger Prokisch

**Affiliations:** 1 Institute of Human Genetics, School of Medicine, Technische Universität München, Munich, Germany.; 2 Institute of Neurogenomics, Helmholtz Zentrum München, Munich, Germany.; 3 Federal State Budgetary Institution of Science “Research Institute of Eye Diseases,” Moscow, Russia.; 4 Department of Neurology, Friedrich-Baur-Institute, University Hospital of the Ludwig-Maximilians-Universität München, Munich, Germany.; 5 Fédération de Génétique et Institut Imagine, Université Paris Descartes, Hôpital Necker Enfants Malades, Paris, France.; 6 Scientific Institute San Raffaele, Milan, Italy.; 7 Genomics Unit, Sheba Cancer Research Center, Sheba Medical Center, Tel-Hashomer, Israel.; 8 Sackler Faculty of Medicine, Tel-Aviv University, Tel-Aviv, Israel.; 9 Wohl Institute for Translational Medicine, Sheba Medical Center, Tel-Hashomer, Israel.; 10 Research Centre for Medical Genetics, Moscow, Russia.; 11 IRCCS Istituto delle Scienze Neurologiche di Bologna, Bologna, Italy.; 12 Oxford Eye Hospital, Oxford University Hospitals NHS Foundation Trust, Oxford, United Kingdom.; 13 Nuffield Laboratory of Ophthalmology, Department of Clinical Neurosciences, University of Oxford, Oxford, United Kingdom.; 14 Department of Pediatrics, Klinikum am Steinenberg, Reutlingen, Germany.; 15 Laboratory Genetics in Ophthalmology (LGO), INSERM UMR1163 - Institute of Genetic Diseases, Imagine. Paris, France.; 16 Unit of Medical Genetics and Neurogenetics, Fondazione IRCCS Istituto Neurologico Carlo Besta, Milan, Italy.; 17 Department of Pathophysiology and Transplantation, University of Milan, Milan, Italy.; 18 Functional Proteomics, Medical School, Goethe University, Frankfurt am Main, Germany.; 19 Institute of Human Genetics, University Medical Center Hamburg-Eppendorf, Hamburg, Germany.; 20 The Hospital for Sick Children, Department of Ophthalmology and Vision Sciences, The University of Toronto, Toronto, Canada.; 21 Department of Neurology, University Hospital Bonn, Bonn, Germany.; 22 Department of Medical Chemistry, Molecular Biology and Pathobiochemistry, Semmelweis University, Budapest, Hungary.; 23 Department of Experimental Epileptology and Cognition Research, University of Bonn, Bonn, Germany.; 24 Unit of Neurology, Department of Biomedical and NeuroMotor Sciences (DIBINEM), University of Bologna, Bologna, Italy.; 25 Bavarian Center for Biomolecular Mass Spectrometry (BayBioMS), Technische Universität München, Munich, Germany.; 26 Institute for Cardiovascular Physiology, Goethe-University Frankfurt, Frankfurt am Main, Germany.; 27 Department of Pediatrics, Salzburger Landeskliniken and Paracelsus Medical University Salzburg, Salzburg, Austria.; 28 Institute for Rare Diseases,; 29 Talpiot Medical Leadership Program, and; 30 Pediatric Neurology Unit, Edmond and Lily Safra Children’s Hospital, Sheba Medical Center, Tel-Hashomer, Israel.; 31 Ophthalmology Department, Centre National d’Ophtalmologie des Qinze-Vingts, Paris, France.; 32 German Center for Neurodegenerative Diseases (DZNE), Munich, Germany.; 33 Munich Cluster of Systems Neurology (SyNergy), Munich, Germany.; 34 German Center for Cardiovascular Research (DZHK), Partner Site RheinMain, Frankfurt, Germany.

**Keywords:** Genetics, Neuroscience, Genetic diseases

## Abstract

Leber’s hereditary optic neuropathy (LHON) is the most frequent mitochondrial disease and was the first to be genetically defined by a point mutation in mitochondrial DNA (mtDNA). A molecular diagnosis is achieved in up to 95% of cases, the vast majority of which are accounted for by 3 mutations within mitochondrial complex I subunit–encoding genes in the mtDNA (mtLHON). Here, we resolve the enigma of LHON in the absence of pathogenic mtDNA mutations. We describe biallelic mutations in a nuclear encoded gene, *DNAJC30*, in 33 unsolved patients from 29 families and establish an autosomal recessive mode of inheritance for LHON (arLHON), which to date has been a prime example of a maternally inherited disorder. Remarkably, all hallmarks of mtLHON were recapitulated, including incomplete penetrance, male predominance, and significant idebenone responsivity. Moreover, by tracking protein turnover in patient-derived cell lines and a *DNAJC30*-knockout cellular model, we measured reduced turnover of specific complex I N-module subunits and a resultant impairment of complex I function. These results demonstrate that DNAJC30 is a chaperone protein needed for the efficient exchange of complex I subunits exposed to reactive oxygen species and integral to a mitochondrial complex I repair mechanism, thereby providing the first example to our knowledge of a disease resulting from impaired exchange of assembled respiratory chain subunits.

## Graphical abstract

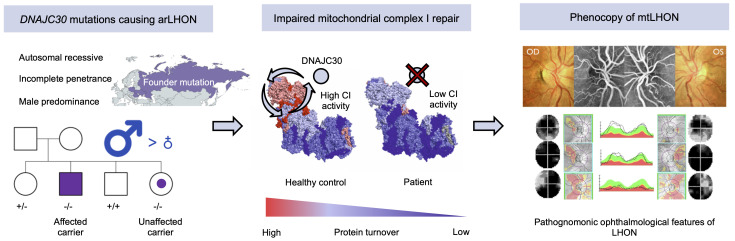

## Introduction

Leber’s hereditary optic neuropathy (LHON, OMIM:535000) was first noted by Von Graefe in 1858 (1) and was formally described as a clinical entity bearing his name by Leber in 1871 (2). LHON results from a rapidly evolving degenerative process and is therefore unique among the hereditary optic atrophies in its clinical course. It presents with subacute, simultaneous or sequential, bilateral painless loss of central vision due to selective degeneration of the retinal ganglion cells (RGCs) and their axons. The disease arises during young adult life and displays a sex-dependent incomplete penetrance of 50% in males and 10% in females (3), resulting in 5 times more males being affected. Permanent severe loss of central vision is the typical endpoint, though spontaneous recovery of visual acuity has occasionally been reported (4, 5, 6).

Though noted in its earliest descriptions to be transmitted through the maternal line (7), the inheritance pattern of LHON was only confirmed over 100 years later by the discovery of the first point mutation in mitochondrial DNA (mtDNA) (m.11778G>A in *MT-ND4*; ref. 8). Along with 2 further point mutations (m.3460G>A in *MT-ND1* and m.14484T>C in *MT-ND6*), altogether these 3 mtDNA mutations account for approximately 90% of LHON cases (mtLHON) with a maternal family history (3, 9, 10). Other rare mtDNA mutations account for a further approximately 5% (11), inclusive of variants associated with MELAS (mitochondrial myopathy, encephalopathy, lactic acidosis, and stroke-like episodes, OMIM:540000; refs. 12, 13, 14) and Leigh syndrome (infantile subacute necrotizing encephalopathy, OMIM:256000; refs. 15, 16).

LHON mutations characteristically disrupt subunits of mitochondrial complex I, the first and largest of the electron transfer complexes, encoded by both nuclear DNA and mtDNA. Complex I is carefully assembled by numerous factors and associates with the other proton translocating complexes to form supercomplexes in the mitochondrial inner membrane. These complexes couple electron flow with proton pumping to generate the membrane potential that drives ATP synthesis (17, 18, 19). To maintain high functionality, complex I subunits exposed to the highest levels of oxidative damage may undergo repair largely independently of the remaining complex, by disassembly and replacement of subunits at a much lower energetic cost than de novo synthesis of the complex in its entirety (20, 21).

## Results

### Identification of pathogenic DNAJC30 mutations in LHON patients in association with a complex I defect.

A homozygous missense variant in *DNAJC30* was initially identified by whole-exome sequencing (WES) in 4 individuals from the German Network for Mitochondrial Disorders (mitoNET), 3 males from 2 families with adult-onset LHON and a female with childhood-onset Leigh syndrome. Through international collaboration within the European Network for Mitochondrial Disorders (GENOMIT), the combined analysis of WES data and Sanger sequencing of further molecularly undiagnosed LHON patients revealed an additional 29 cases associated with *DNAJC30* mutations. mtDNA screening failed to detect any other pathogenic variants or rare variants of uncertain significance (VUS) (Supplemental Table 1; supplemental material available online with this article; https://doi.org/10.1172/JCI138267DS1). Moreover, in those investigated by WES, no biallelic pathogenic variants or VUS were detected in nuclear genes encoding complex I subunits or assembly factors (Supplemental Table 2).

**Figure 1:**
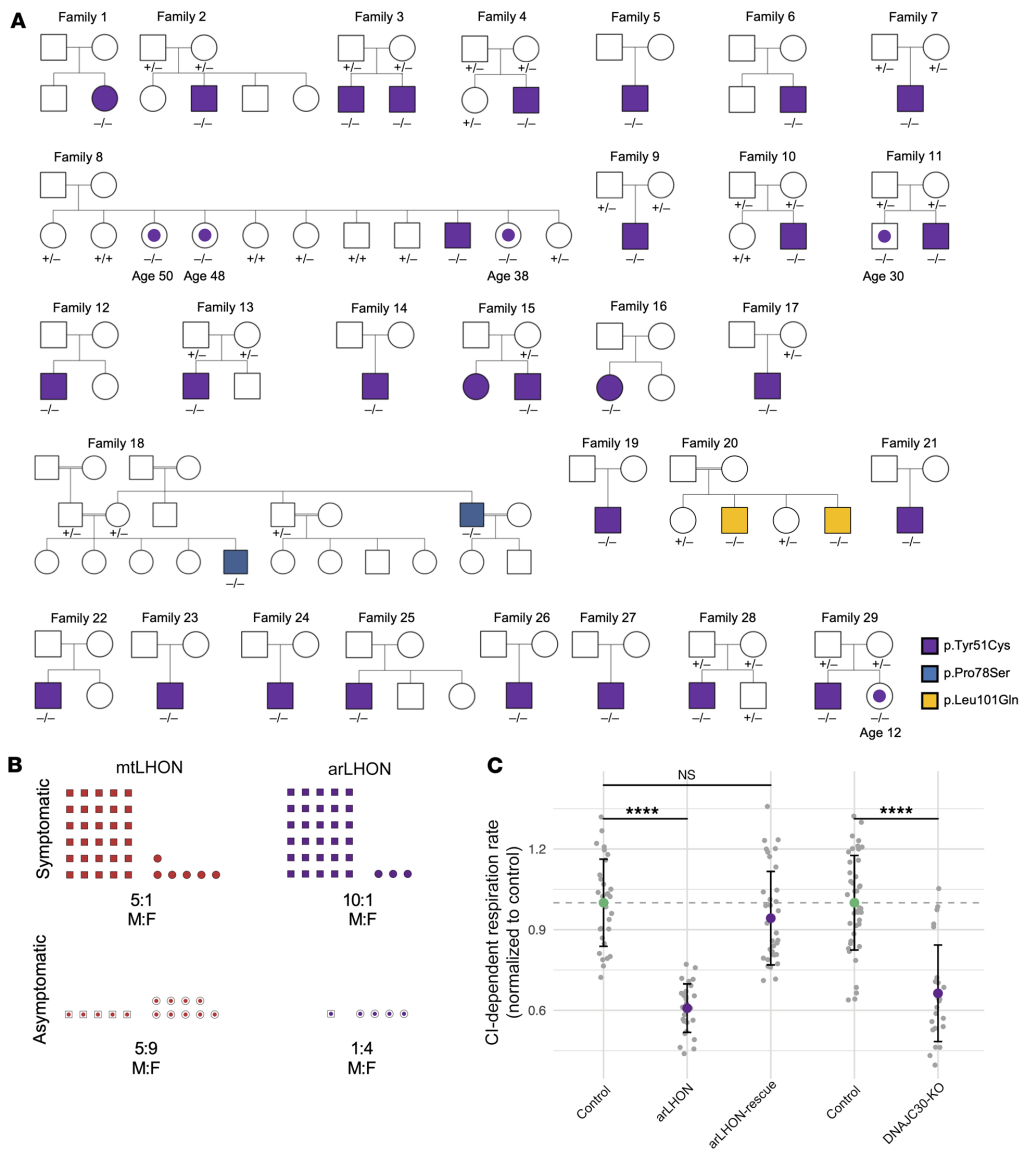
Identification of pathogenic *DNAJC30* mutations in LHON patients in association with a complex I defect. (**A**) Pedigrees from 29 families. The genotype is denoted by (–/–) for homozygous variant carriers, (+/–) for heterozygous variant carriers, and (+/+) for carriers of 2 wild-type alleles. Individuals with a central dot are homozygous carriers (–/–) who did not express the disease phenotype at their current age, stated beneath. (**B**) Schematic of the sex-dependent incomplete penetrance in both maternally inherited LHON (mtLHON) and recessive LHON (arLHON), demonstrating a clear male predominance in symptomatic carriers of disease-causing variants. (**C**) Mitochondrial complex I–dependent (CI-dependent) respiration rate measurement in control (*n =* 30, technical replicates) and arLHON (*n =* 28, technical replicates) fibroblast cell lines, demonstrating a mild respiratory defect rescued by reexpression of naive DNAJC30 (arLHON-rescue, *n =* 36, technical replicates). The defect in CI-dependent respiration rate is recapitulated in the *DNAJC30*-KO (*n =* 25, technical replicates) in comparison with control (*n =* 43, technical replicates) HEK293 cell lines. Data are normalized to the respective control cell line and depicted by the mean ± SD; 2-sided Student’s *t* test, *P* values corrected for multiple comparisons to the control (Dunnett’s test). ****P ≤ 0.0001. NS, not significant.

Of the 33 subjects, 29 harbored the same NM_032317.2 c.152A>G (7:73097602, NP_115693.2 p.Tyr51Cys) homozygous variant with an allele frequency of 0.125% (351:281136 alleles, no homozygous carriers) in gnomAD (https://gnomad.broadinstitute.org/). The remaining 4 patients, from Family 18 and Family 20, harbored an NM_032317.2 c.232C>T (7:73097522, NP_115693.2, p.Pro78Ser) and an NM_032317.2 c.302T>A (7:73097452, NP_115693.2, p.Leu101Gln) homozygous variant, respectively, both of which are absent in gnomAD (Figure 1A).

Due to the recessive mode of inheritance, and therefore the absence of a family history, identification of pedigrees with unaffected homozygous carriers is challenging. Despite this, our collection of pedigrees indicates incomplete penetrance, with 5 homozygous mutation carriers not expressing a phenotype. In total, 30 of 31 (96.8%) homozygous carrier males and just 3 of 7 (42.9%) homozygous carrier females express a phenotype. This results in a significant male predominance, with a 10:1 ratio of affected males to affected females (*P <* 0.001, Fisher’s exact test; Figure 1B). The penetrance of the *DNAJC30* variants can only be approximated, and is unquestionably an overestimate due to the limited number of large pedigrees and unaffected siblings of differing carrier status in our study. Nevertheless, these observations resemble the incomplete penetrance associated with mtLHON, in which penetrance is reported to be approximately 50% in males and 10% in females. This results in a 5:1 ratio of affected males to affected females (refs. 3, 22; and Figure 1B). To investigate the underlying reason for the higher penetrance in males, we measured DNAJC30 expression on the RNA and protein level in over 75 and 100 controls, respectively; however, we did not detect a significant difference (Supplemental Figure 1, A and B). DNAJC30 availability is therefore not the factor influencing sex-dependent variable penetrance. In mtLHON, the mtDNA population-specific polymorphisms of haplogroup J are proposed play a modifying role in penetrance, as patients with the haplogroup J genetic background demonstrate higher penetrance (23). This association is recapitulated in LHON with an autosomal recessive mode of inheritance (arLHON), as 5 of 27 investigated arLHON patients (18.5%) have the haplogroup J genetic background, which is greater than the expected 9% for the European population (ref. 24 and Table 1).

Given that the majority of the patients originate from Russia, Poland, Romania, and Ukraine, the p.Tyr51Cys *DNAJC30* variant is ascribed to an Eastern European founder event. The variant is estimated to have arisen 85 generations ago (95% CI 43–168 generations) based on the genetic length of ancestral haplotypes shared between individuals (Supplemental Figure 1C). Taking the allele frequency of the variant (0.125%) and applying the Hardy-Weinberg equation with adjustment for the observed incomplete penetrance, arLHON is estimated to affect 1.09 per million individuals, a figure just below the established prevalence estimate of LHON once adjusted for the prior assumption that up to 95% are accounted for by mtDNA mutations (1.61 per million, 95% CI 1.24–1.99 per million; ref. 25). Furthermore, in Russia, the contribution of *DNAJC30* variants to the heritability of LHON is considerably higher. Of 86 molecularly confirmed LHON patients investigated by full mtDNA sequencing and WES in the Research Centre for Medical Genetics in Moscow, 18 (20.9%) harbored the homozygous p.Tyr51Cys *DNAJC30* mutation, while 68 (79.1%) harbored pathogenic mtDNA mutations. Male prominence was demonstrated in both forms of LHON (arLHON 17:1 affected male to affected female ratio, mtLHON 15:2 affected male to affected female ratio). Additional evidence for incomplete penetrance was provided by screening of the Research Centre for Medical Genetics institutional database of WES data from 1036 patients with inherited disease, where we found 10 heterozygous carriers of the p.Tyr51Cys variant, indicating a minor allele frequency of 0.48% in the founder population. This predicts 2.3 per 100,000 homozygous carriers. If the variant were fully penetrant, this figure would exceed the expected number of LHON cases, thereby strengthening the argument for incomplete penetrance. The marked incomplete penetrance in females is supported by an equal allele frequency in male and female individuals of the p.Tyr51Cys founder variant in the gnomAD database (male 0.12%, female 0.13%).

All 3 *DNAJC30* variants occur in a conserved area of the protein, the J domain (Supplemental Figure 1D). This domain belongs to the heat shock protein (HSP) family of chaperones and is key to their functional interactions (26). The p.Tyr51Cys and p.Leu101Gln variants are positioned in close proximity on the protein structure (Supplemental Figure 1E) and lead to degradation of the protein (Supplemental Figure 1F). These amino acids are therefore likely to be fundamental to the structural integrity of the protein. The p.Pro78Ser variant, which does not lead to protein degradation (Supplemental Figure 1F), is predicted to disrupt function due to its position in a crucial functional region, the His, Pro, and Asp tripeptide (HPD; ref. 26 and Supplemental Figure 1D).

Consistent with mtLHON, a complex I defect was invariably demonstrated on skeletal muscle biopsy in investigated arLHON patients (Supplemental Figure 1G). Complex IV and complex V activity was not disrupted (Supplemental Figure 1, H and I). Analyzing complex I–dependent respiration in patient-derived fibroblast cell lines (*n =* 7) revealed a significant defect (mean 69% of control, SD 16%, *P <* 0.001), which is modest yet consistent across patients and genotypes and is in keeping with, if not more subtle than, the magnitude of the complex I defect seen in mtDNA LHON patient–derived fibroblast cell lines (*n =* 3; Supplemental Figure 1, J and K). The complex I–dependent defect in respiration was rescued by reexpression of naive *DNAJC30* in the patient cell line (Figure 1C). Moreover, the complex I–dependent respiration defect was recapitulated in the *DNAJC30-*KO HEK293 cell line (mean 66% of control, SD 18%, *P <* 0.0001; Figure 1C).

### LHON associated with DNAJC30 mutations presents as a phenocopy of maternally inherited LHON.

**Figure 2:**
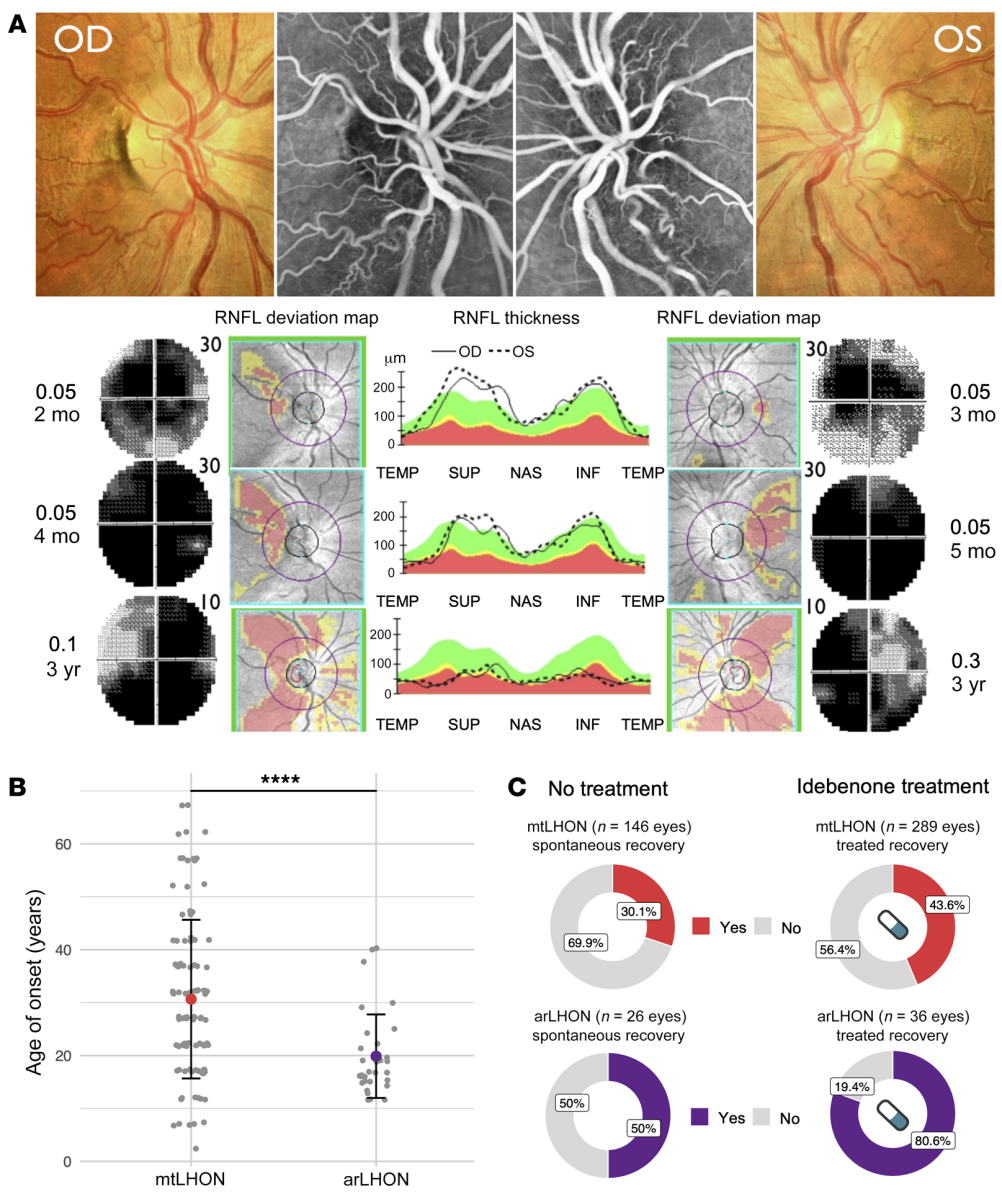
LHON associated with *DNAJC30* mutations presents as a phenocopy of maternally inherited LHON. (**A**) The pathognomonic triad of ophthalmological features in mtLHON is recapitulated in arLHON. Presented here is an illustrative example from 1 arLHON patient. Top panel: Optic nerve head picture and fluorescein angiography in the acute stage of the disease displaying microangiopathy without leakage, fiber swelling, and initial temporal pallor of the optic disc. Bottom panel: The retinal nerve fiber layer (RNFL) thickness analysis and deviation map showing the progressive thinning of fibers from the subacute stage (right eye [OD] at 2 months and left eye [OS] at 3 months after visual loss) to the chronic stage (3 years). The 30° Humphrey visual field shows progressive enlargement of the central scotoma in the subacute stage (from 2 to 4 months in OD and from 3 to 5 months in OS) and fenestration of the scotoma after 3 years (10° Humphrey visual field) associated with recovery of visual acuity (VA, expressed in decimal units). m, months; y, years. RNFL thickness (middle) is displayed as a function of the quadrant in the deviation map: temporal (TEMP), superior (SUP), nasal (NAS), and inferior (INF). (**B**) Age of onset (years) in mtLHON (*n =* 104) (28) and arLHON (*n =* 31). Data presented as mean ± SD. ****P ≤ 0.0001 by 2-sided Student’s *t* test. (**C**) Spontaneous and idebenone-treated rate of clinically significant recovery of VA, defined as improvement in logMAR VA of ≥0.2, in mtLHON (4, 5, 6) in comparison with arLHON, in which treated recovery rates were significantly higher in arLHON (mtLHON 43.6%, arLHON 80.6%, *P <* 0.001, Fisher’s exact test).

From a clinical perspective, physicians representing national centers of expertise in LHON were unable to stratify the LHON patients by mtDNA or nuclear origin. The pathognomonic triad of ophthalmological features: (a) circumpapillary telangiectatic microangiopathy, (b) vessel tortuosity of the central retinal vessels without leakage on fluorescein angiography, and (c) the hallmark, subacute phase swelling (pseudoedema) of the retinal nerve fiber layer (RNFL) (27), were documented in all arLHON subjects (Table 2 and exemplified in Figure 2A). In all arLHON subjects the subacute phase was followed by an atrophic chronic phase with generalized thinning of the RNFL due to RGC and axonal degeneration. No macular or peripheral retinal abnormalities were reported, and brain magnetic resonance imaging (MRI) was reportedly normal in 19 of 22 investigated arLHON patients. There was no record of extraocular manifestations. In this regard, the clinical phenotype of arLHON is an indistinguishable phenocopy of mtLHON (see Supplemental Methods for individual patient case reports).

The statistical analysis uncovered subtle differences both in the age of onset and recovery rate of visual acuity in arLHON in comparison with mtLHON. The age of onset (mean 19.9 ± 7.9 [SD] years) was earlier and more condensed than that reported for mtLHON (mean 30.7 ± 15.0 [SD] years, *P <* 0.001; ref. 28, Table 1, and Figure 2B). Age-related expression of DNAJC30 on the RNA and protein level measured in control fibroblast cell lines was unable to provide an explanation for this pattern of onset (Supplemental Figure 2, A and B). The median time from involvement of the first eye to involvement of the second eye was 1 week (range 0 to 2 years), and 14 patients (46.7%) demonstrated bilateral involvement at onset. The median time from onset to nadir was 8 weeks (range 0 to 2 years; Table 2). We acknowledge that the natural history of the cohort may be confounded to some extent by the concurrent use of idebenone in the majority of the cases.

Clinically relevant recovery (CRR) of visual impairment from nadir, defined as improvement in logMAR (logarithm of the minimum angle of resolution) visual acuity (VA) of 0.2 or greater (29), was observed in 42 eyes (67.7%, in 22 patients). In 8 eyes (12.9%, in 6 patients) VA recovery was complete (Table 2). Idebenone therapy was received by 18 patients, as a potent antioxidant and electron donor approved for LHON by EMA (https://www.ema.europa.eu/en/medicines/human/EPAR/raxone), bypassing complex I to restore downstream mitochondrial electron flow and respiration (29). Of the 36 treated and 26 untreated eyes, CRR was reported in 29 (80.6%) and 13 (50.0%), respectively. The treated recovery was significantly higher than that reported for mtLHON (refs. 4–6; *P <* 0.001, Fisher’s exact test, Figure 2C). The mean time from onset to first CRR was 13.0 ± 10.4 (SD) months, and 25.8 months ± 30.3 (SD) months, in the treated and untreated eyes, respectively. This is comparable to mtLHON, in which the mean time from onset to first CRR is reported to be 17.2 ± (SD) 7.8 months in idebenone-treated cases, and 27.7 ± (SD) 22.5 months in idebenone-naive cases (5).

We also report 1 patient with Leigh syndrome in the absence of optic involvement. This is a female patient, presenting at 2 years of age with spasticity, dysarthria, disturbance of gait, a moderate lactate peak on magnetic resonance spectroscopy (MRS), and bilateral necrosis of the putamen with lesions in the pedunculi cerebelli suggestive of Leigh syndrome on brain MRI (see Supplemental Methods for the case report).

### DNAJC30 mutations result in impaired exchange of specific subunits of mitochondrial complex I.

Due to the consistent complex I defect found in the patient muscle biopsies, we analyzed quantitative proteomic data from patient-derived (*n =* 3) and control (*n =* 105) fibroblast cell lines, to determine whether DNAJC30 defects lead to a decrease in complex I abundance. However, to our surprise, differential expression analysis (DEA) followed by gene set enrichment analysis for 149 mitochondrial pathways (MitoPathways3.0; https://www.broadinstitute.org/files/shared/metabolism/mitocarta/human.mitocarta3.0.path_.html) demonstrated a subtle yet significant increase of 12.1% in “Complex I subunits” in the patient cell lines (adjusted *P* = 0.04, based on mean log_2_[fold change]; Supplemental Figure 3A and Supplemental Tables 3 and 4). Given the impaired complex I–dependent respiration in patient-derived fibroblast cell lines, this subtle increase in complex I abundance results in low relative specific activity of complex I (*P <* 0.0001; Supplemental Figure 3B). There was no significant signal for any other OXPHOS complex to indicate a general increase in mitochondrial biogenesis (Supplemental Figure 3A and Supplemental Tables 3 and 4). The specificity of the DNAJC30 defect for complex I was also confirmed by a subtle yet significant increase in complex I in a quantitative proteomic analysis of detergent dodecyl maltoside–solubilized (DDM-solubilized) respiratory chain complexes (RCCs) resolved by blue native electrophoresis (BNE) in the *DNAJC30*-KO HEK293 cell line (*P* = 0.0002; Supplemental Figure 3C). The abundance of the remaining RCCs was not significantly increased (complex III *P* = 0.38, complex IV *P* = 0.50).

To determine whether complex I was properly assembled, we investigated a patient-derived fibroblast cell line and the *DNAJC30*-KO HEK293 cell line by complexome profiling. Here, we found intact and correctly assembled complex I with no assembly intermediates in association with defective DNAJC30 (Supplemental Figure 3, D and E). Together with the quantitative proteomic data, these data confirmed that DNAJC30 is not a subunit or assembly factor of complex I, which would be expected to decrease the abundance and disrupt the assembly of the complex. The complexome data also recapitulate loss of the DNAJC30 protein due to the p.Tyr51Cys founder mutation, as demonstrated in our earlier quantitative proteomic approach (Supplemental Figures 1F and 3D), and confirmed the successful knockout of DNAJC30 in the *DNAJC30*-KO HEK293 cell line (Supplemental Figure 3E). Moreover, in the complexome profiling of the control fibroblast and control HEK293 cell lines, DNAJC30 was found to run at the size of the complex I–containing supercomplex, where it is present in substoichiometric quantities (1:200 DNAJC30/complex I), indicating a transient interaction with the complex I–containing supercomplex (Supplemental Figure 3, D and E, and Supplemental Table 5). The association of DNAJC30 with the complex I–containing supercomplex was supported across 6 independent quantitative proteomic experiments of digitonin-solubilized RCCs resolved by BNE in our study and by multiple previously published complexome data sets across human and mouse samples, as summarized in Supplemental Table 6.

Although the quantitative proteomic data in patient-derived fibroblast cell lines indicates a subtle increase in complex I subunit abundance due to DNAJC30 defect, RNA sequencing did not reveal a corresponding increase in complex I subunit expression, analyzed by a DEA of 149 mitochondrial pathways (MitoPathways3.0) between patient-derived fibroblast (*n =* 3) and control fibroblast (*n =* 79) cell lines (count data, Supplemental Table 7; complete DEA results, Supplemental Tables 8 and 9). This indicated that there may be a problem in the degradation of complex I subunits. We therefore measured the turnover of single proteins within mitochondrial protein complexes by combining pulse stable isotope labeling of amino acids in cell culture (pSILAC) with mass spectrometry of assembled RCCs resolved by BNE (see Methods).

**Figure 3:**
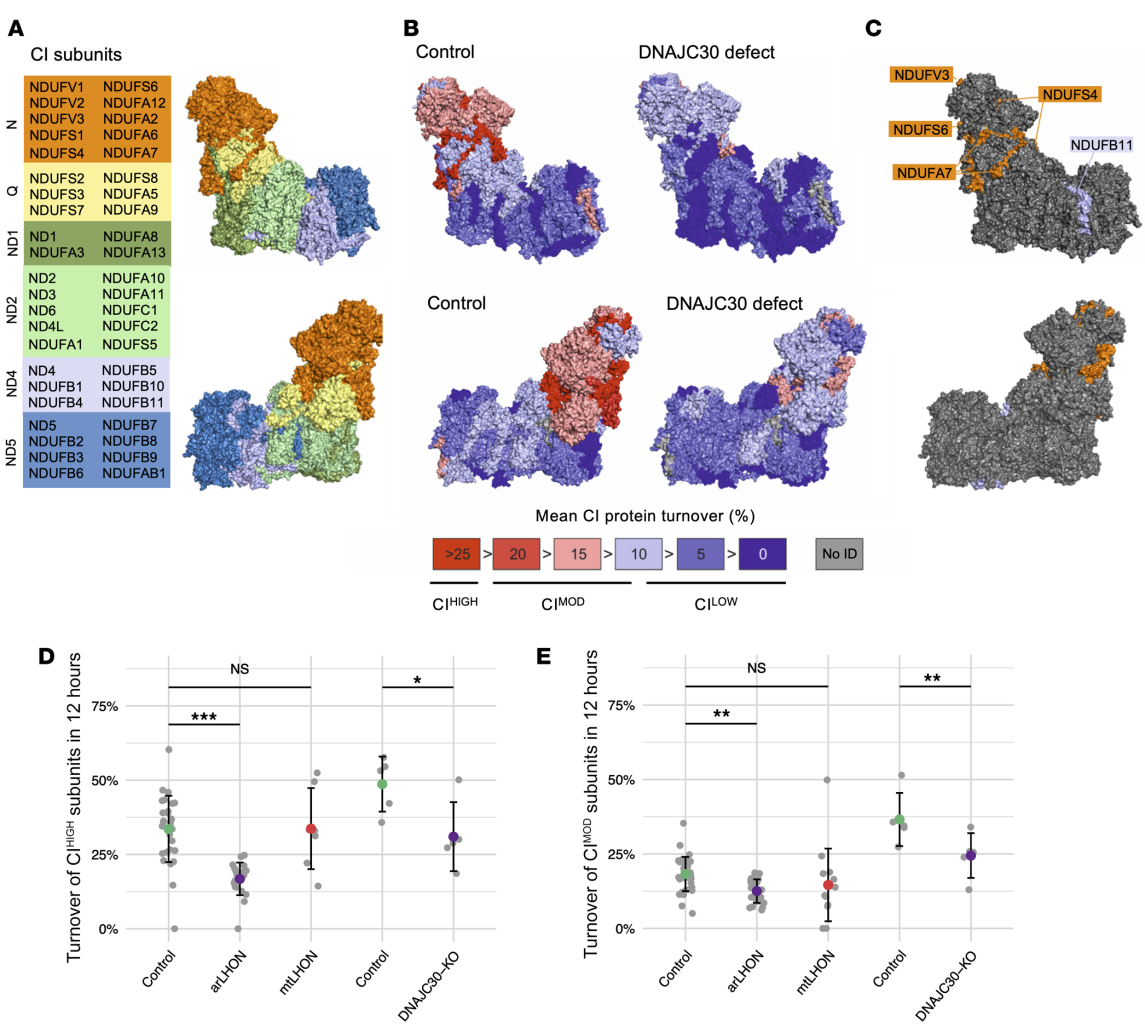
*DNAJC30* mutations result in impaired repair of specific subunits of mitochondrial complex I. (**A**) The structure of mitochondrial complex I (CI) (30), depicted by module and respective protein. (**B**) Mitochondrial CI structure colored by the mean degree of subunit turnover in 12 hours in control fibroblast cell lines (*n =* 7) and patient fibroblast cell lines (*n =* 6) depicted as a percentage. The mean data are provided in Supplemental Table 15 and the individual experiments are depicted in Supplemental Tables 11–14. (**C**) The DNAJC30 interacting partners in CI according to the BioPlex database highlighted on the CI structure. The interaction partners in the N-module (NDUFV3, NDUFS4, NDUFS6, and NDUFA7) account for 4 of the 5 CI^HIGH^ subunits, defined as subunits with >25% turnover in 12 hours in the control fibroblast cell lines. (**D**) Turnover measurement of CI^HIGH^ subunits (*n =* 5) and (**E**) CI^MOD^ subunits (*n =* 5) in 12 hours in control (*n =* 7), arLHON (*n =* 6), and mtLHON patient (*n =* 3, m.3460G>A in *MT-ND1*, m.11778G>A in *MT-ND4*, and m.14484T>C in *MT-ND6*) fibroblast cell lines, and control (*n =* 1) and *DNAJC30*-KO (*n =* 1) HEK293 cell lines. arLHON patients demonstrate a defect in CI^HIGH^ (control mean 33.6% ± 11.2% SD, patient mean 16.8% ± 5.5% SD) and CI^MOD^ (control mean 18.3% ± 5.7% SD, patient mean 12.5% ± 3.9% SD) subunits. The defective turnover of CI^HIGH^ subunits is shown to be specific to arLHON (CI^HIGH^ subunits, control mean 33.6% ± 11.2% SD, mtLHON mean 33.7% ± 13.7% SD). The *DNAJC30*-KO HEK293 cell line demonstrates a defect in CI^HIGH^ (control mean 48.7% ± 8.3% SD, KO mean 31.0% ± 11.6% SD) and CI^MOD^ (control mean 36.6% ± 8.9% SD, KO mean 24.5% ± 7.5% SD) subunits. Data depicted as the mean ± SD; 2-sided Student’s *t* test, *P* values corrected for multiple comparisons to the control (Dunnett’s test). *P ≤ 0.05, **P ≤ 0.01, ***P ≤ 0.001. NS, not significant. A complete summary of the data is provided in Supplemental Table 10 and the experiment is depicted in Supplemental Tables 10–15 and 17.

It is known from recent studies that subunits of the complex I N-module (Figure 3A and ref. 30) require exchange, due to exposure to higher levels of oxidative damage (20, 21). This results in higher turnover rates of the N-module subunits that are independent of the rest of the assembled complex. In the analysis of control fibroblast cell lines (*n =* 7), we confirmed the presence of differential rates of turnover in the individual subunits of complex I (Figure 3B and Supplemental Tables 10–15). Moreover, we can further subdivide the complex I subunits into 3 categories according to their respective turnover rates, as CI^HIGH^ and CI^MOD^ — together accounting for the N-module of complex I — and CI^LOW^, representing the remainder of complex I. The CI^HIGH^ subunits (NDUFV3, NDUFS4, NDUFS6, NDUFA6, and NDUFA7) demonstrate a mean turnover of greater than 25% in 12 hours in control fibroblast cell lines (mean 33.6% ± 11.2% SD), while the CI^LOW^ subunits — accounting for the complex I Q-, ND1-, ND2-, ND4-, and ND5-module subunits — have a mean turnover of 8.7% (± 6.0% SD). Each of these CI^HIGH^ subunits has been identified as a late participant in complex I assembly according to Guerrero-Castillo et al. (31), indicating that these proteins are less stably bound within the complex and might readily be exchangeable. The CI^MOD^ subunits, accounting for the remainder of the complex I N-module subunits, have a mean turnover of 18.3% (± 5.7% SD).

The patient-derived fibroblast cell lines (*n =* 6), spanning all 3 *DNAJC30* mutations, demonstrated a significant decrease in the turnover of N-module subunits in assembled complex I (Figure 3B and Supplemental Tables 10–15). The mean turnover of the CI^HIGH^ subunits was most strongly reduced to 16.8% from 33.6% (*P <* 0.0001) followed by the CI^MOD^ subunits to 12.5% from 18.3% (*P <* 0.001) (Figure 3, B–E, and Supplemental Tables 10–15). In explanation of this finding, consultation of the BioPlex database of protein-protein interactions confirmed 4 out of the 5 CI^HIGH^ proteins to be the direct interaction partners of DNAJC30, across 5 independent experiments (Figure 3C, complete results in Supplemental Table 16; and refs. 32, 33). Together, these data indicate that the interaction of DNAJC30 with specific complex I subunits facilitates the high turnover of complex I N-module subunits in a complex I repair mechanism. This defect was not found in mtLHON (*n =* 3) and is therefore specific to arLHON (Figure 3, D and E). The defect shows no predilection to sex to account for the difference in sex-dependent penetrance (Supplemental Figure 3, F and G).

To further validate these findings, and to question the specificity of the defect to complex I, we studied the protein turnover of more than 1,200 proteins in the control and *DNAJC30*-KO HEK293 cell line, detecting 56 proteins with high turnover, defined as greater than 25% at 12 hours. The data recapitulate the defect seen in the patient-derived fibroblast cell lines, with a mean turnover in CI^HIGH^ subunits of 31.0% in the *DNAJC30*-KO, in comparison with 48.7% in the control (*P* = 0.013; Figure 3D). This was also seen in the CI^MOD^ subunits, with a mean turnover of 24.5% in the *DNAJC30*-KO, in comparison with 36.6% in the control (*P* = 0.002; Figure 3E and Supplemental Table 17). In contrast, there was no DNAJC30-specific difference in the turnover of other complex I subunits, all other OXPHOS subunits (including those with high turnover), and all other detected proteins with high turnover (Supplemental Figure 3H). Moreover, by the analysis of differential protein turnover within protein complexes (see Supplemental Methods), we verified that among all captured protein complexes (*n =* 145), only mitochondrial complex I, and specifically the complex I N-module, had significantly altered turnover due to the knockout of *DNAJC30* (mean delta turnover, 14.3% ± 7.6% SD, adjusted *P* = 0.04; Supplemental Table 18). Interestingly, among the top 6 mitochondrial proteins of similar phylogenetic profile to DNAJC30 are CLPX, CLPB, and HSP70 (HSPA9), components of the mitochondrial protein degradation machinery (Supplemental Table 19 and ref. 34).

**Figure 4:**
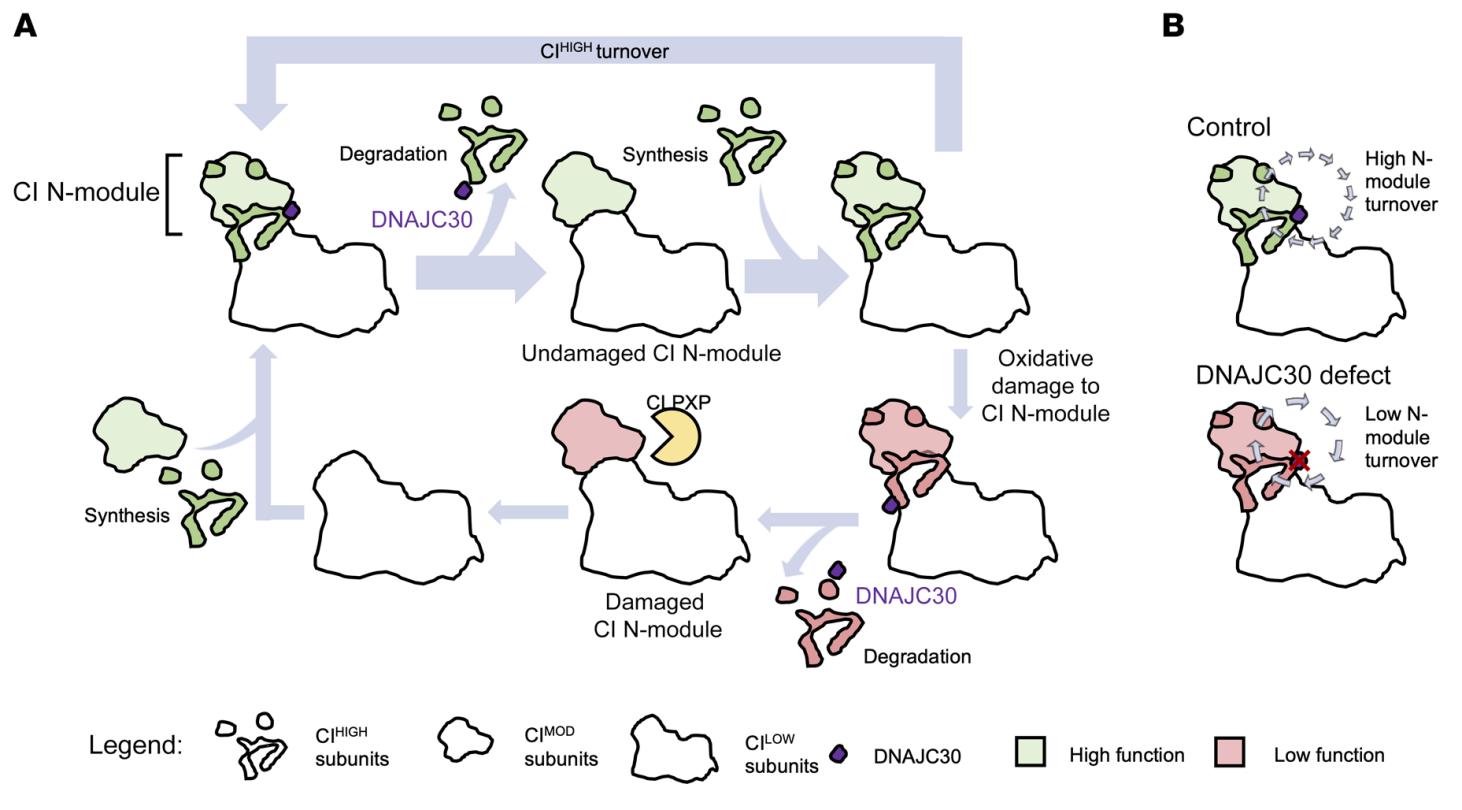
Schematic representation of the proposed role of DNAJC30 in complex I repair. (**A**) In normal physiological conditions, DNAJC30 interacts with specific complex I (CI) N-module proteins (CI^HIGH^), facilitating their disassembly and subsequent degradation. In the setting of highly functional CI, these proteins are newly synthesized and replaced without degradation of further subunits. In the case of oxidative damage to the CI N-module, upon disassembly of the CI^HIGH^ subunits by DNAJC30 the protease CLPXP may access and remove the damaged CI^MOD^ subunits (20, 21). Along with the CI^HIGH^ subunits, these subunits are subsequently resynthesized and replaced, negating the need for complete degradation and synthesis of CI at high energetic cost. (**B**) Compared with control, in the presence of *DNAJC30* mutations turnover of the N-module subunits is decreased, impairing the CI repair mechanism and leading to the accumulation of CI with reduced function.

We thus propose DNAJC30 to be a chaperone protein involved in the exchange of CI^HIGH^ subunits (NDUFV3, NDUFS4, NDUFS6, NDUFA6, and NDUFA7), facilitating the protein degradation machinery, CLPXP, to access and degrade the N-module subunits exposed to higher levels of oxidative damage, and collectively maintaining complex I with high function (Figure 4A). This functional link between DNAJC30 and CLPXP is supported by the evolutionary co-occurrence of both proteins. In contrast, in the absence of DNAJC30 the complex I N-module subunits may no longer be readily exchangeable, resulting in an accumulation of complex I with lower function, which is also reported in defects of the CLPXP subunits (ref. 20, 21; and Figure 4B).

## Discussion

Here we describe a recessive phenocopy of mtLHON due to mutations in the nuclear gene *DNAJC30*, where we observe incomplete penetrance and male predominance, phenomena seldom occurring in recessive disease. The evidence supporting these observations derives from the high allele frequency of the founder mutation and the equal distribution of the allele between males and females in gnomAD, which should otherwise result in a common disease of equal sex distribution in the setting of full penetrance. Our data therefore indicate that the factor(s) driving incomplete penetrance and male predominance originate downstream of the primary genetic event, secondary to subtle anatomical, hormonal, or otherwise physiological discrepancies between sexes, or due to other genetic or epigenetic factors. Given that all investigated patient WES data sets were negative for rare biallelic variants in genes encoding complex I subunits and assembly factors, and for rare mtDNA variants, we could not attribute the incomplete penetrance to a modifying factor at play in these genomic regions. Therefore, akin to mtLHON, the factor influencing sex-dependent penetrance remains elusive at this time. Cautious interpretation of penetrance is required, both due to the relatively limited number of families from which to draw conclusions, and due to reports of the LHON phenotype manifesting up to the eighth decade of life (35). However, given that the vast majority of patients with arLHON express the disease in the second to third decade of life, and that the current age of almost all asymptomatic homozygous variant carriers is within the range of 30 to 50 years, it is expected that they will continue to be asymptomatic. A future epidemiological study would be necessary to extensively explore this matter.

We provide molecularly undefined LHON patients with a diagnosis and demonstrate a further example of Leigh syndrome within an LHON/MELAS/Leigh syndrome spectrum. This also occasionally arises in specific rare mtDNA mutations affecting mtDNA-encoded subunits of complex I (15, 16). In contrast to the infrequently described nuclear DNA abnormalities with “LHON-like” visual loss, such as in patients with Charcot-Marie-Tooth disease, in optic atrophy associated with dominant mutations in *OPA1* (36), and in a single report of recessive “LHON-like” optic neuropathy due to mutations in *NDUFS2* (37), our findings argue for the frequent occurrence of arLHON. This is effectively exemplified at the Research Centre for Medical Genetics in Moscow, where the p.Tyr51Cys *DNAJC30* founder mutation accounts for over 20% of molecularly diagnosed LHON patients. We therefore recommend parallel sequencing of the complete mtDNA sequence and the 1-exon gene *DNAJC30* to close the diagnostic gap in LHON. Moreover, as arLHON is associated with an alternative mode of inheritance, an earlier age of onset, and a marked benefit from treatment with idebenone, even somewhat better than patients with mtLHON, reaching a genetic diagnosis has valuable implications in prognostic counseling and treatment decisions.

In arLHON, the mitochondrial complex I defect characteristic of the disease results from the impaired exchange of specific complex I N-module subunits exposed to higher risk of oxidative damage. The exchange of these subunits occurs at a lower energetic cost than complete de novo replacement of the complex and maintains high functionality (20, 21, 38, 39). The subtlety of the complex I–dependent respiration defect in the patient-derived fibroblast cell lines reflects the role of DNAJC30 in maintenance rather than in the structure or assembly of complex I, akin to a factor optimizing complex I to ensure full bioenergetic function. The interaction of DNAJC30 with complex I ascribes to the protein an additional function to that suggested to date, in which DNAJC30 has been shown to play a role in mitochondrial and neuronal function and morphology by interaction with the ATP-synthase machinery (complex V) to facilitate ATP synthesis (40). Complementary to our study of defective DNAJC30 in patient-derived fibroblast cell lines and in the *DNAJC30*-KO HEK293 cellular model, Tebbenkamp and colleagues demonstrated increased complex I–containing supercomplexes and hypofunctional mitochondria with significantly reduced respiration rate in cultured primary neurons from a *Dnajc30*-KO mouse model. Our data, however, did not demonstrate defective DNAJC30 to affect complex V function in the patient muscle biopsies, complex V abundance on proteomic analyses, nor complex V subunit turnover. Clinically, defects in DNAJC30 have previously been suggested to contribute to the pathogenesis of William’s syndrome, a 7q11.23 hemideletion of 26 to 28 genes inclusive of *DNAJC30*, which akin to LHON includes diminished neuronal function (40). However, both homozygous and heterozygous *DNAJC30* variant carriers in our study displayed no clinical features of William’s syndrome. Moreover, gnomAD reports 25 heterozygous loss-of-function variant carriers in addition to 351 heterozygous p.Tyr51Cys variant carriers, which is also confirmed in our study to result in loss of the protein, comparable to a hemizygous deletion, in a reportedly healthy population. This argues that defects in DNAJC30, though potentially enhancing the phenotype of the haploinsufficiency of the genes within the William’s syndrome locus, is alone unable to recapitulate all elements of the phenotype.

To conclude, we resolve the enigma of the LHON phenotype in the absence of any pathogenic mtDNA mutations, and describe biallelic mutations of a nuclear DNA–encoded gene, *DNAJC30*, in so far unsolved LHON patients. This argues for “LHON” as a clinical description independent of transmission. Given this, we would suggest LHON to be subdivided as mtLHON and arLHON, due to the important impact on genetic counseling. With a combination of incomplete penetrance and higher than expected allele frequency, the discovery of this disease gene proved challenging to current diagnostic approaches. Moreover, we unveil the pathomechanism of recessive LHON and gain insight into a biological pathway. We demonstrate DNAJC30 to be a chaperone protein integral to a complex I repair mechanism, controlling the exchange of complex I subunits likely damaged by exposure to reactive oxygen species. As dysfunctional complex I is the most frequent biochemical feature of mitochondrial disease (41, 42) and is broadly implicated in the pathogenesis of cancer, diabetes, parkinsonism, and aging (43, 44, 45), this study highlights DNAJC30 as a target within the complex I repair pathway with potential therapeutic implications far beyond the optic neuropathies.

## Methods

### Study subjects.

Clinical diagnosis of LHON was established by experienced ophthalmologists and neurologists specialized in mitochondrial disease across institutions in Germany, Italy, the United Kingdom, France, Russia, Canada, and Israel. The clinical features of each patient are presented in Tables 1 and 2. Detailed case reports are presented in the Supplemental Methods.

### Cell lines and tissue culture.

Patient-derived fibroblast cell lines were obtained by skin biopsy from the responsible clinician with written informed consent. Normal human dermal fibroblasts (NHDFs) were obtained from Lonza. HEK293 cells were obtained from Thermo Fisher Scientific. All cell lines were grown in Dulbecco’s Modified Eagle Medium (DMEM, Life Technologies), supplemented with 10% fetal bovine serum (FBS), 1% penicillin/streptomycin, and 200 μM uridine. The culture medium was replaced once every 2–3 days. Cell lines were grown at 37°C in the presence of 5% CO_2_. All patient-derived fibroblast cell lines tested negative for mycoplasma contamination. Selective growth of the *DNAJC30*-transfected cell line was maintained by the supplementation of 5 μg/mL blasticidin to the cell culture medium. Selective growth of the *DNAJC30*-KO HEK293 cell line was maintained by the addition of 0.5 μg/mL puromycin to the cell culture medium.

### Generation of the DNAJC30-KO HEK293 cell line.

The commercially available Origene KN2.0 non–homology-mediated CRISPR knockout kit was utilized according to the manufacturer’s instructions. Biallelic knockout of *DNAJC30* resulted from donor integration in the first allele and a c.158_159insA (NM_032317.2) frameshift mutation in the second allele leading to a premature stop codon within the J domain (p.Leu53Leufs*58, NP_115693.2) and complete loss of the protein on proteomic analyses.

### BNE.

Sample preparation and BNE of cultured cell pellets were essentially done as described previously (46). Briefly, cells were collected by scraping and centrifugation, further disrupted using a precooled motor-driven glass/Teflon Potter-Elvehjem homogenizer at 2000 rpm and 40 strokes. Homogenates were centrifuged for 15 minutes at 600*g* to remove nuclei, cell debris, and intact cells. Mitochondrial membranes were sedimented by centrifugation of the supernatant for 15 minutes at 22,000*g*. Mitochondria-enriched membranes from 20 mg cells were resuspended in 35 μL solubilization buffer (50 mM imidazole pH 7, 50 mM NaCl, 1 mM EDTA, 2 mM aminocaproic acid) and solubilized with 10 μL 20% digitonin (Serva) or 5 μL 20% DDM. Samples were supplemented with 2.5 μL 5% Coomassie G250 in 500 mM aminocaproic acid and 5 μL 0.1% Ponceau S in 50% glycerol. Equal amounts of sample protein were loaded on top of a 3% to 18% acrylamide gradient gel (dimension 14 × 14 cm). After native electrophoresis in a cold chamber, blue-native gels were fixed in 50% (v/v) methanol, 10% (v/v) acetic acid, and 10 mM ammonium acetate for 30 minutes and stained with Coomassie (0.025% Serva Blue G, 10% [v/v] acetic acid).

### Mass spectrometry of assembled RCCs resolved by BNE.

Procedures and parameters for mass spectrometry and data analysis are summarized at the PRIDE-proteomics identification database (https://www.ebi.ac.uk/pride/) with the identifiers PXD021385, PXD021386, PXD021500, PXD022340, PXD022339, and PXD021548.

### pSILAC metabolic labeling.

For pSILAC metabolic labeling, medium was changed into medium containing ^13^C_6_,^15^N_4_-L-arginine and ^13^C_6_,^15^N_2_-L-lysine (Silantes) for 0, 6, 8, 10, and 12 hours. Mitochondrial membranes were solubilized with either digitonin (Patient runs 1, 2, 4, and HEK293 run, as per Supplemental Table 10) or DDM (Patient run 3, as per Supplemental Table 10), protein complexes resolved by BNE and the band(s) representing either the supercomplex and complex V or the individual complexes, after digitonin and DDM solubilization respectively, were used for mass spectrometry. For protein turnover the ratio of heavy intensity–based absolute quantification (IBAQ)/sum of light and heavy IBAQ was calculated. Pymol version 2.3.3 (https://pymol.org/2/) was used to generate the turnover heatmap for the mouse complex I structure (PDB 6g2; ref. 30).

### Additional information.

WES was performed as described previously (37, 47, 48). RNA sequencing was performed as described in Kremer et al. (47). Complementation of patient-derived fibroblast cell lines was performed by lentivirus-mediated expression of the full-length *DNAJC30* cDNA using the ViraPower HiPerform Lentiviral TOPO Expression Kit (Thermo Fisher Scientific) according to that described previously (49). Measurement of oxygen consumption rate (complex I–dependent respiration rate) was performed as described previously (49). All other methods and data analyses appear in the Supplemental Methods.

### Statistics.

All statistical analyses were performed with R version 1.1.423. The choice of statistical test was determined by visual inspection of the distribution of the data. Where assumptions of normality and equal variance were met, the parametric 2-sided Student’s *t* test was used to compare the means of 2 groups. When multiple comparisons were made to a control, the *P* values were corrected with Dunnett’s test. Categorical data were analyzed by Fisher’s exact test. The statistical test is stated in the respective figure legend. All reported *P* values are 2-tailed with Bonferroni’s correction for multiple testing. *P ≤* 0.05 was considered statistically significant. The *P* values are annotated in the figures by *P ≤ 0.05, **P ≤ 0.01, ***P ≤ 0.001, and ****P ≤ 0.0001.

### Study approval.

This study was in accordance with the ethical principles of the Declaration of Helsinki and received local ethical committee approval from each center for their respective patient(s). Written informed consent was obtained from all patients, parents, or legal guardians as required.

### Data availability.

The proteomics data are available in ProteomeXchange (http://www.proteomexchange.org/) via the PRIDE (50) partner repository with the identifiers PXD021385, PXD021386, PXD021500, PXD022340, PXD022339, PXD021548, and PXD021499.

## Author contributions

HP conceived and designed the study. SLS and HP wrote the manuscript and all authors edited the manuscript. SLS, TDK, WSK, C Lamperti, and IW performed experiments. SLS and IW analyzed results. CBC, NAA, ZA, PB, OB, IB, LC, M Capristo, M Carbonelli, MLC, PCI, PF, SG, DG, JH, MH, EH, YSI, EJ, JK, RK, CK, RKN, TDK, WSK, CLM, C Lamperti, C Ludwig, PFM, AM, JAM, JM, TAN, FP, BPS, MSS, TMS, FT, MT, ATV, CVC, MW, EYZ, NVZ, JMR, VC, PGT, NLS, RB, EG, and TK provided essential materials. HP, IW, TK, DG, CL, and VC provided funding and supervision.

## Supplementary Material

Supplemental data

Supplemental Tables 1-19

## Figures and Tables

**Table 2:**
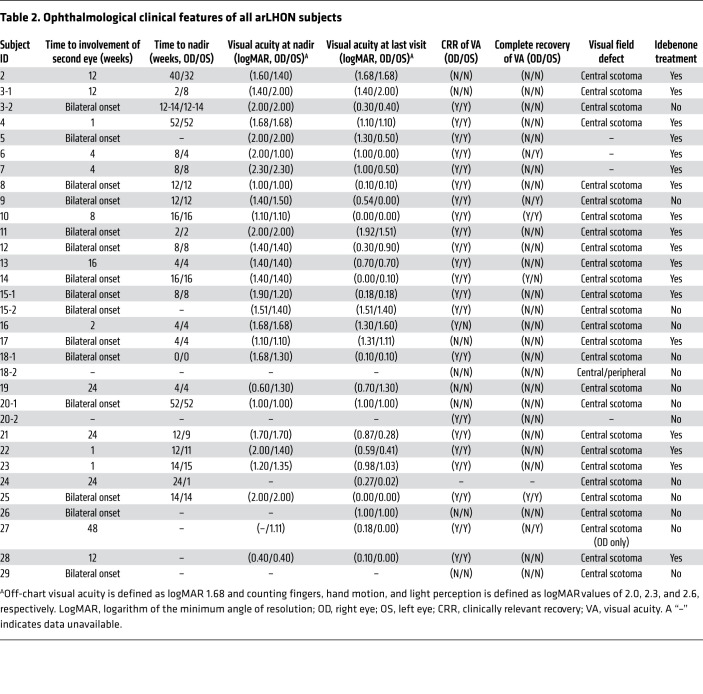
Ophthalmological clinical features of all arLHON subjects

**Table 1:**
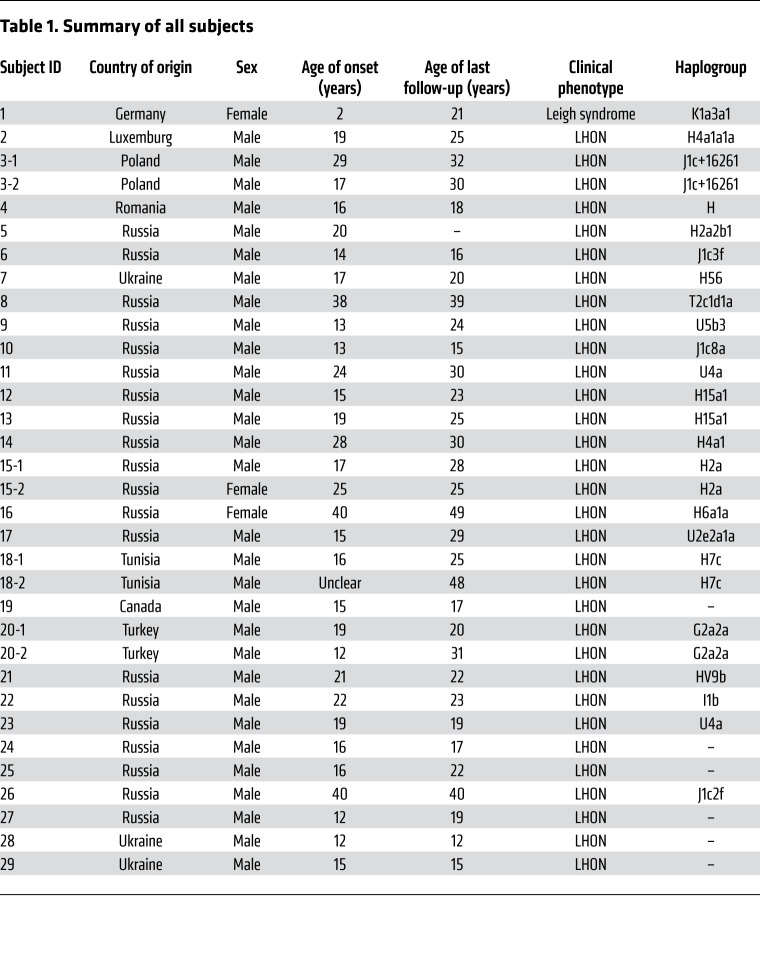
Summary of all subjects

## Version 1 01/19/2021

In-Press Preview

## Version 2 03/15/2021

Electronic publication
