# Supplementary material for: Impaired complex I repair causes recessive Leber’s hereditary optic neuropathy
Source: J Clin Invest. 2021 Mar 15;131(6):e138267. doi: 10.1172/JCI138267 (PMC7954600; doi:10.1172/JCI138267)
Supplement: Supplemental data [file jci-131-138267-s056.pdf]

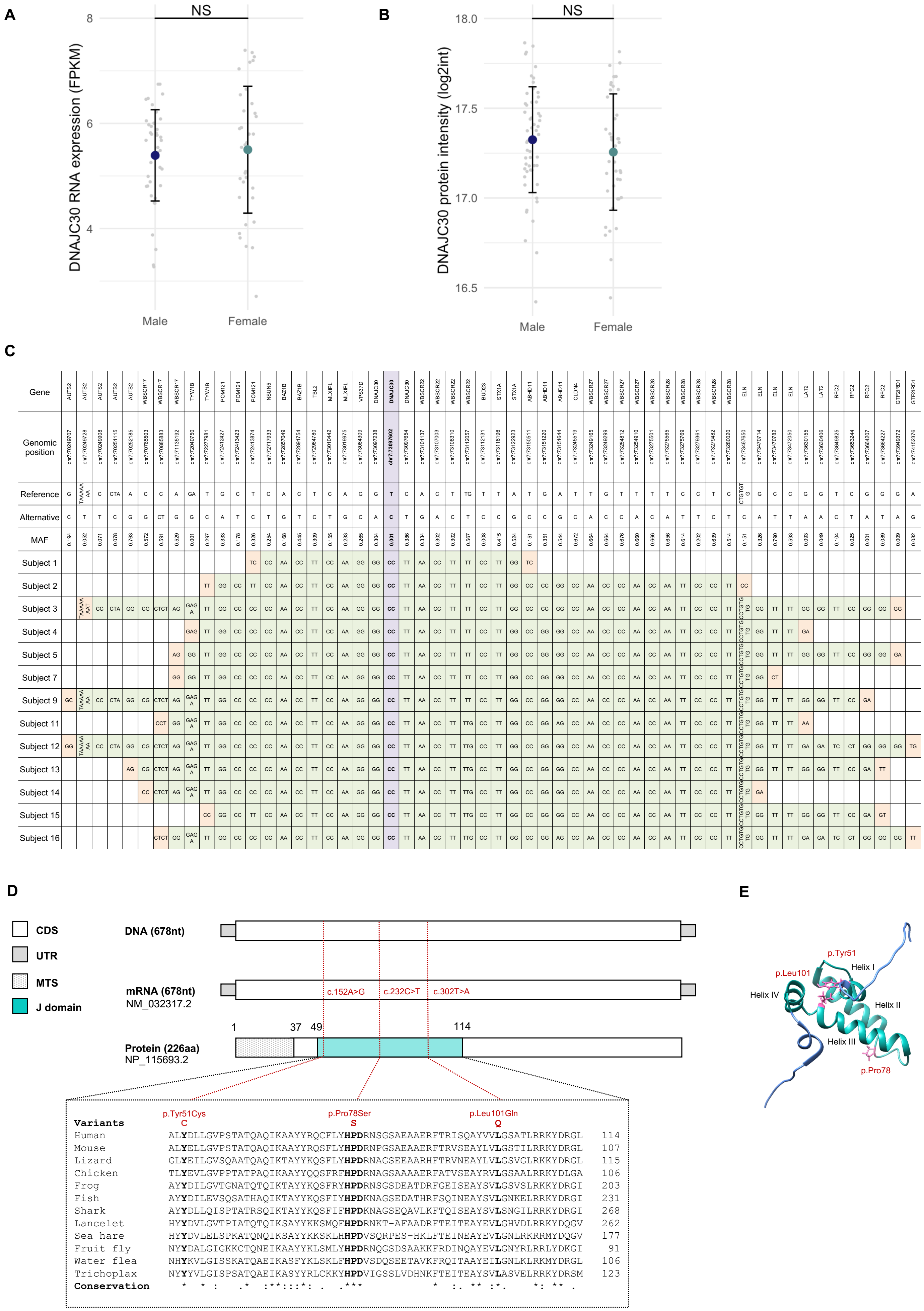
A
NS
DNAJC30 RNA expression (FPKM)
8
6
4
Male
Female
B
NS
DNAJC30 protein intensity (log2int)
18.0
17.5
17.0
16.5
Male
Female
C
Gene
Genomic position
Reference
Alternative
MAF
Subject 1
Subject 2
Subject 3
Subject 4
Subject 5
Subject 7
Subject 9
Subject 11
Subject 12
Subject 13
Subject 14
Subject 15
Subject 16
D
CDS
UTR
MTS
J domain
DNA (678nt)
mRNA (678nt)
NM_032317.2
c.152A>G
c.232C>T
c.302T>A
Protein (226aa)
NP_115693.2
1
37
49
114
p.Tyr51Cys
p.Pro78Ser
p.Leu101Gln
Variants C S Q
Human ALYDLLGVPSTATQAQIKAAYYRQCFLYHPDRNSGSAEAAERFTRISQAYVVLGSATLRRKYDRGL 114
Mouse ALYELLGVPSTATQAQIKAAYYRQSFLYHPDRNPGSAEAAERFTRVSEAYLVLGSTILRRKYDRGL 107
Lizard GLYEILGVSQAATQAQIKTAYYKQSFLYHPDRNAGSEEAARHFTRVNEAYLVLGSVGLRKKYDRGL 115
Chicken TLYEVLGVPPTATPAQIKAAYYQQSFRFHPDRNAGSAAAAERFATVSEAYRVLGSAALRRKYDLGA 106
Frog AYYDILGVTGNATQTQIKTAYYKQSFRYHPDRNSGSDEATDRFGEISEAYSVLGSVSLRKKYDRGI 203
Fish AYYDILEVSQSATHAQIKTAYYKQSFIYHPDKNAGSEDATHRFSQINEAYSVLGNKELRRKYDRGI 231
Shark AYYDLLQISPTATRSQIKTAYYKQSFIFHPDKNAGSEQAVLKFTQISEAYSVLSNGILRRKYDRGI 268
Lancelet HYYDVLGVTPIATQTQIKSAYYKKSMQFHPDRNKT-AFAADRFTEITEAYEVLGHVDLRRKYDQGV 262
Sea hare HYYDVLELSPKATQNQIKSAYYKKSKLHHPDVSQRPES-HKLFTEINEAYEVLGNLRKRRMYDQGV 177
Fruit fly NYYDALGIGKKCTQNEIKAAYYKLSMLYHPDRNQGSDSAAKKFRDINQAYEVLGNYRLRRLYDKGI 91
Water flea NHYKVLGISSKATQAEIKASFYKLSKLFHPDVSDQSEETAVKFRQITAAYEILGNLKLRKMYDRGL 106
Trichoplax NYYYVLGISPSATQAEIKASYYRLCKKYHPDVIGSSLVDHNKFTEITEAYSVLASVELRRKYDRSM 123
Conservation * * : .* :**:::*: . .*** * :. ** :*. *: ** .
E
p.Tyr51
p.Leu101
Helix I
Helix IV
Helix II
Helix III
p.Pro78

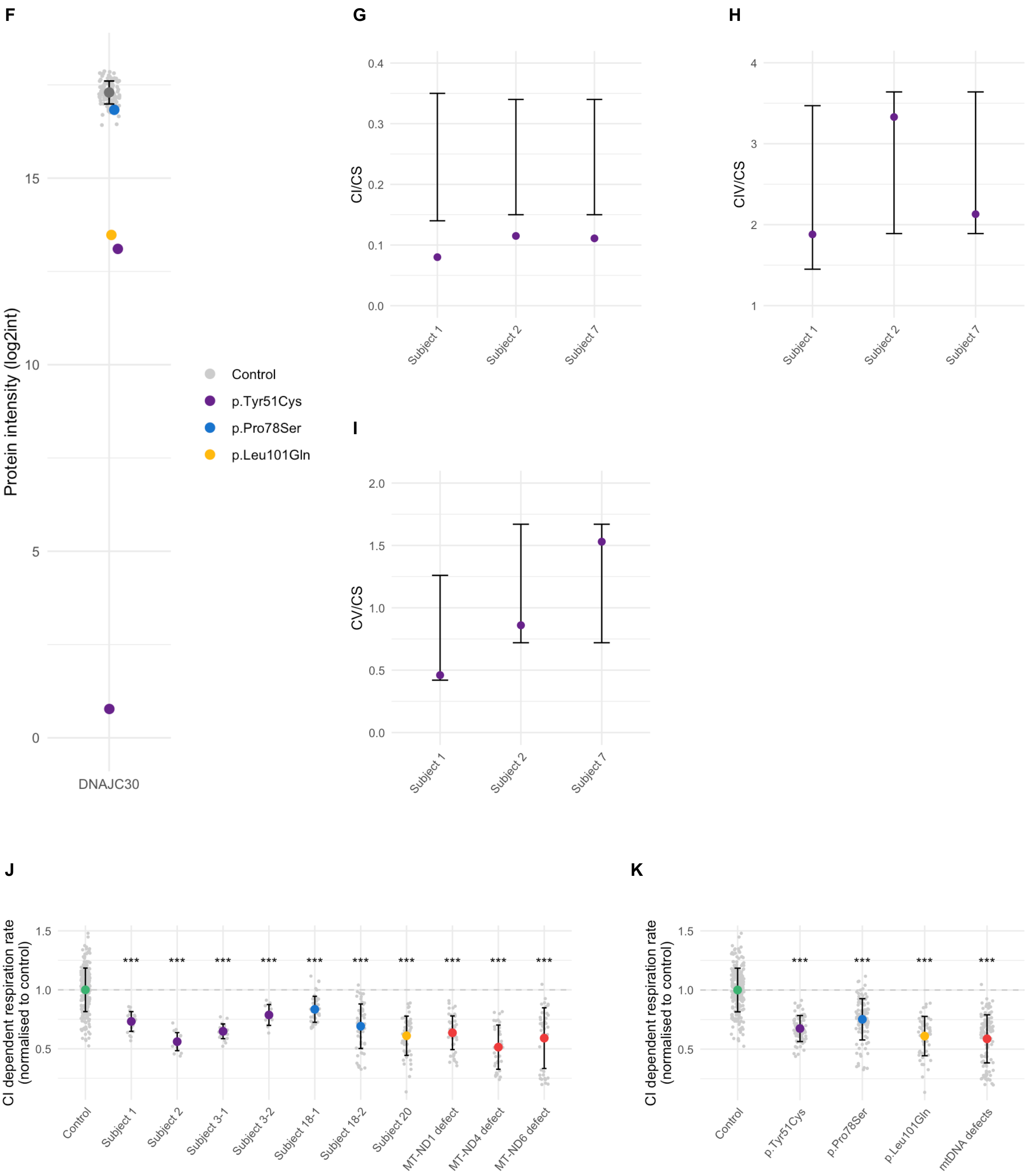

F
Protein intensity (log2int)
0
5
10
15
DNAJC30
Control
p.Tyr51Cys
p.Pro78Ser
p.Leu101Gln
G
CI/CS
0.0
0.1
0.2
0.3
0.4
Subject 1
Subject 2
Subject 7
H
CIV/CS
1
2
3
4
Subject 1
Subject 2
Subject 7
I
CV/CS
0.0
0.5
1.0
1.5
2.0
Subject 1
Subject 2
Subject 7
J
CI dependent respiration rate (normalised to control)
0.5
1.0
1.5
***
Control
Subject 1
Subject 2
Subject 3-1
Subject 3-2
Subject 18-1
Subject 18-2
Subject 20
MT-ND1 defect
MT-ND4 defect
MT-ND6 defect
K
CI dependent respiration rate (normalised to control)
0.5
1.0
1.5
***
Control
p.Tyr51Cys
p.Pro78Ser
p.Leu101Gln
mtDNA defects

Supplementary Figure 1. DNAJC30 expression on RNA and protein level, DNAJC30 mutations on the DNA, mRNA, and protein structure, and DNAJC30 associated CI defect.

(A) *DNAJC30* RNA expression (FPKM) from transcriptomic analysis of male (n=42) and female (n=37) control fibroblast cell lines. These data demonstrate no gender dependent difference in the expression of *DNAJC30*. Data are depicted by the mean ± s.d., two-sided Student's t-test. **(B)** DNAJC30 protein intensity from TMT-labeled quantitative proteomics of male (n=60) and female (n=45) control fibroblast cell lines. These data demonstrate no gender dependent difference in the expression of DNAJC30. Data are depicted by the mean ± s.d., two-sided Student's t-test. **(C)** Founder status of the *DNAJC30* p.Tyr51Cys variant. SNP genotypes are shown in the proximity of the *DNAJC30* p.Tyr51Cys variant (purple) in probands homozygous for the variant. Ancestral haplotypes shared between individuals carrying the mutation ranged in length from 0.4-3.7 Mb (green). Heterozygous or homozygous deviations (orange) from the prevailing genotype indicate recombination occurring outside of these genetic lengths. Using these shared genetic lengths, we estimate the mutation to have occurred approximately 85 generations ago, using the method developed by (1) **(D)** Schematic representation of the *DNAJC30* gene, *DNAJC30* mRNA, and DNAJC30 protein. Pathogenic variants (depicted in red) fall within the highly conserved J domain. The alignment is displayed across 12 species with the human DNAJC30 amino acid sequence as reference. The positions of pathogenic variation and the HPD tripeptide are emboldened. CDS: coding sequence; UTR: untranslated region; MTS: mitochondrial targeting sequence. **(E)** 2YUA yeast model of the DNAJC30 protein from amino acid position 39-124 on the human sequence. The amino acids subject to pathogenic variation in the patients are depicted in red. The p.Tyr51 and p.Leu101 amino acids are seen to be in close proximity and are likely to be integral to the structure of the protein. The p.Pro78 amino acid falls in the HPD tripeptide between helix II and helix III, key to the functional interactions of DNAJC30. **(F)** DNAJC30 protein intensity (log2int) measured by TMT-labeled quantitative proteomics in control (n=105), p.Tyr51Cys (n=2), p.Pro78Ser (n=1), and p.Leu101Gln (n=1) fibroblast cell lines. The mean of the control samples is depicted ± s.d. Both the p.Tyr51Cys and p.Leu101Gln variant lead to degradation of the resultant protein (2.3% and 7.3% DNAJC30 protein remaining, respectively), while the protein resulting from the p.Pro78Ser variant falls within range of the control samples and is likely to exert pathogenicity by interruption of the HPD functional domain (72.9% DNAJC30 protein remaining). **(G)** Mitochondrial CI measurement in skeletal muscle biopsy reveals a consistent CI defect across three investigated patients. **(H)** Mitochondrial CIV measurement in skeletal muscle biopsy reveals no defect. **(I)** Mitochondrial CV measurement in skeletal muscle biopsy reveals no defect. In (G-I) the patient measurement is depicted by a point with the laboratory reference range depicted by a black bar. All values are normalized to the respective citrate synthase (CS) measurement. **(J)** Mitochondrial CI dependent respiration rate measurement in patient-derived fibroblast cell lines (control n=189, Subject 1 n=20, Subject 2 n=17, Subject 3-1 n=22, Subject 3-2 n=12, Subject 18-1 n=42, Subject 18-2 n=57, Subject 20 n=50, MT-ND1 defect n=34, MT-ND4 defect n=48, and MT-ND6 defect n=30, technical replicates). Data are depicted by the mean ± s.d., two-sided Student's t-test, p values corrected for multiple comparisons to the control (Dunnett's test). **(K)** Mitochondrial CI dependent respiration rate measurement summarized from (J) in control and patient fibroblast cells lines by genotype (control n=189, p.Tyr51Cys n=71, p.Pro78Ser n=99, and p.Leu101Gln n=50, technical replicates), demonstrating a mild respiratory defect consistent across *DNAJC30* genotypes comparable to the mtDNA defects responsible for mtLHON. Data are depicted by the mean ± s.d., two-sided Student's t-test, p values corrected for multiple comparisons to the control (Dunnett's test).

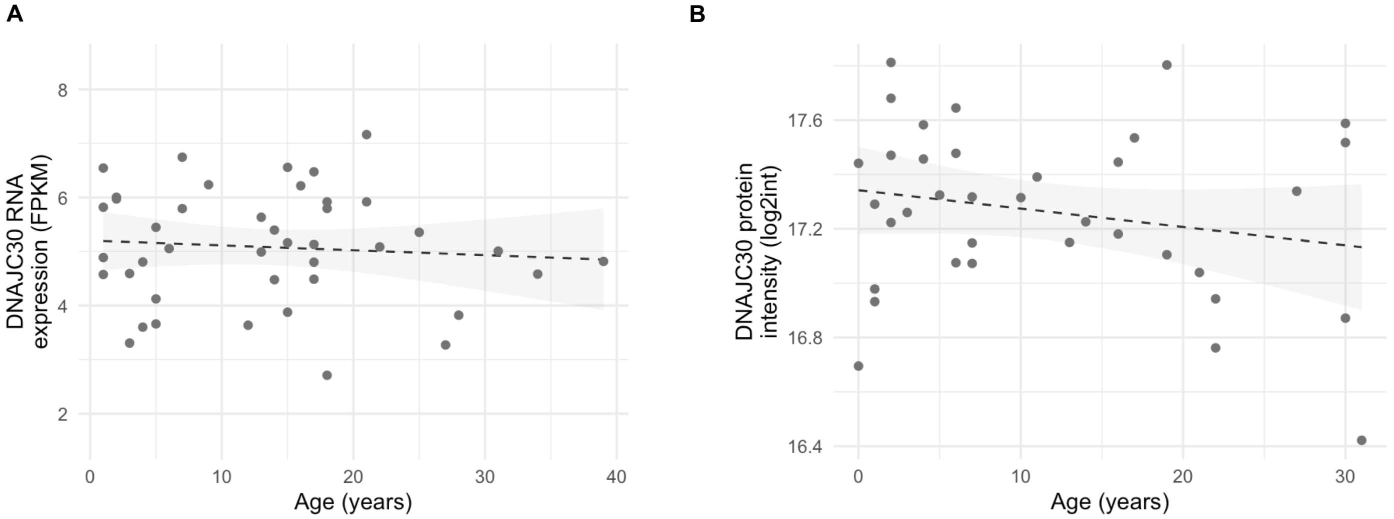

Supplementary Figure 2. Age effect on DNAJC30 expression.

(a) *DNAJC30* expression (FPKM) in fibroblast cell lines from controls of differing age at skin biopsy (years) (n=42). Data are depicted with the 95% confidence interval, Pearson correlation coefficient *R* -0.08, p 0.60. These data do not provide an explanation for the sudden onset of arLHON in the 2nd to 3rd decade of life. (b) DNAJC30 protein intensity (log2int) measured by TMT-labeled quantitative proteomics in fibroblast cell lines from controls of differing age at skin biopsy (n=36). Data are depicted with the 95% confidence interval, Pearson correlation coefficient *R* -0.22, p 0.20. These data do not provide an explanation for the sudden onset of arLHON in the 2nd to 3rd decade of life.

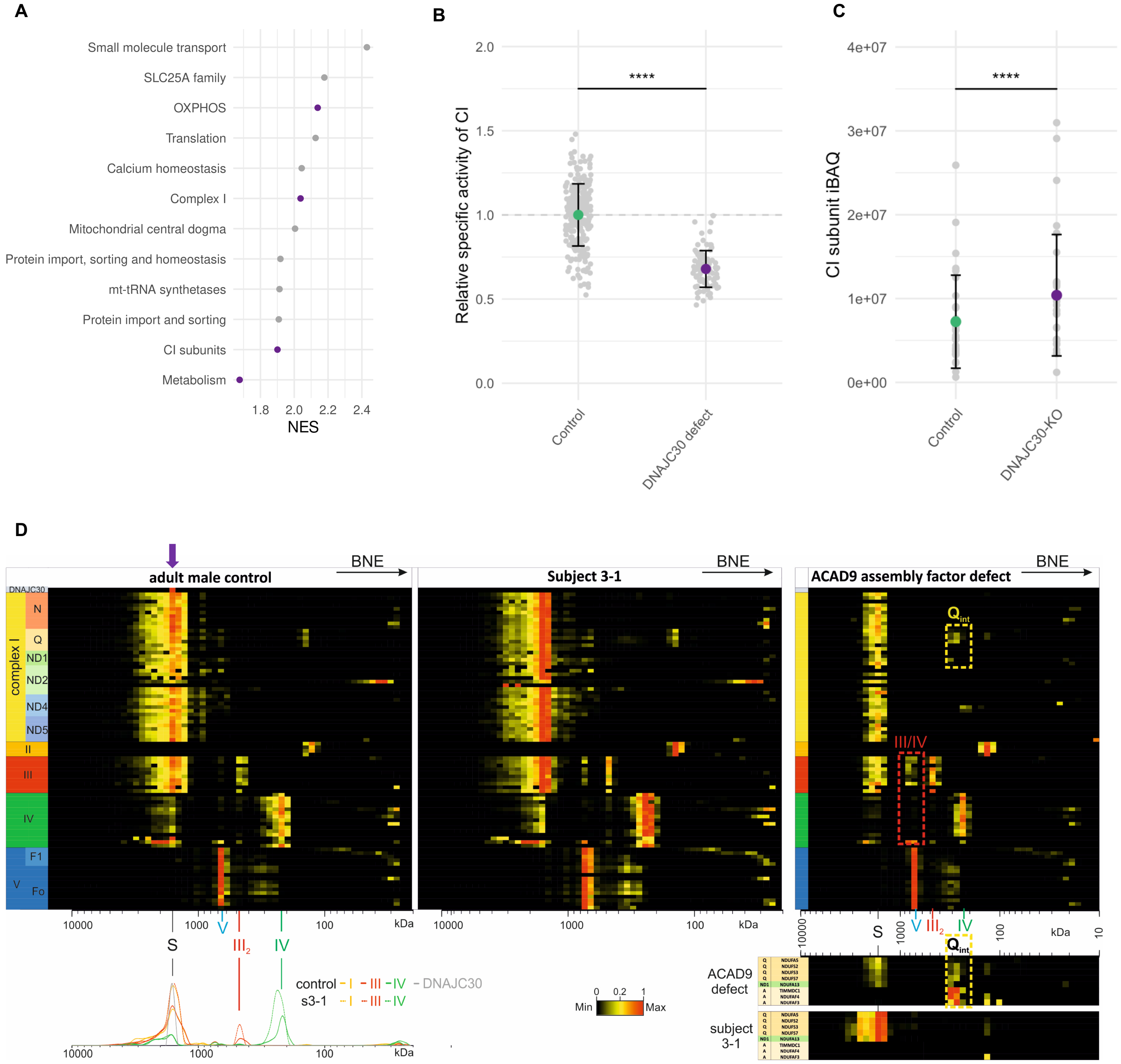
A
Small molecule transport
SLC25A family
OXPHOS
Translation
Calcium homeostasis
Complex I
Mitochondrial central dogma
Protein import, sorting and homeostasis
mt-tRNA synthetases
Protein import and sorting
CI subunits
Metabolism
1.8 2.0 2.2 2.4
NES
B
Relative specific activity of CI
2.0 1.5 1.0 0.5 0.0
****
Control
DNAJC30 defect
C
CI subunit iBAQ
4e+07 3e+07 2e+07 1e+07 0e+00
****
Control
DNAJC30-KO
D
adult male control
BNE
Subject 3-1
ACAD9 assembly factor defect
DNAJC30
complex I
N
Q
ND1
ND2
ND4
ND5
II
III
IV
F1
V
Fo
Qint
III/IV
10000 1000 100 kDa 10
S
V
III2
IV
control
s3-1
DNAJC30
Min
Max
0 0.2 1
ACAD9 defect
subject 3-1
NDUFA5
NDUFS2
NDUFS3
NDUFS7
NDUFA13
TIMMDC1
NDUFAF4
NDUFAF3

E

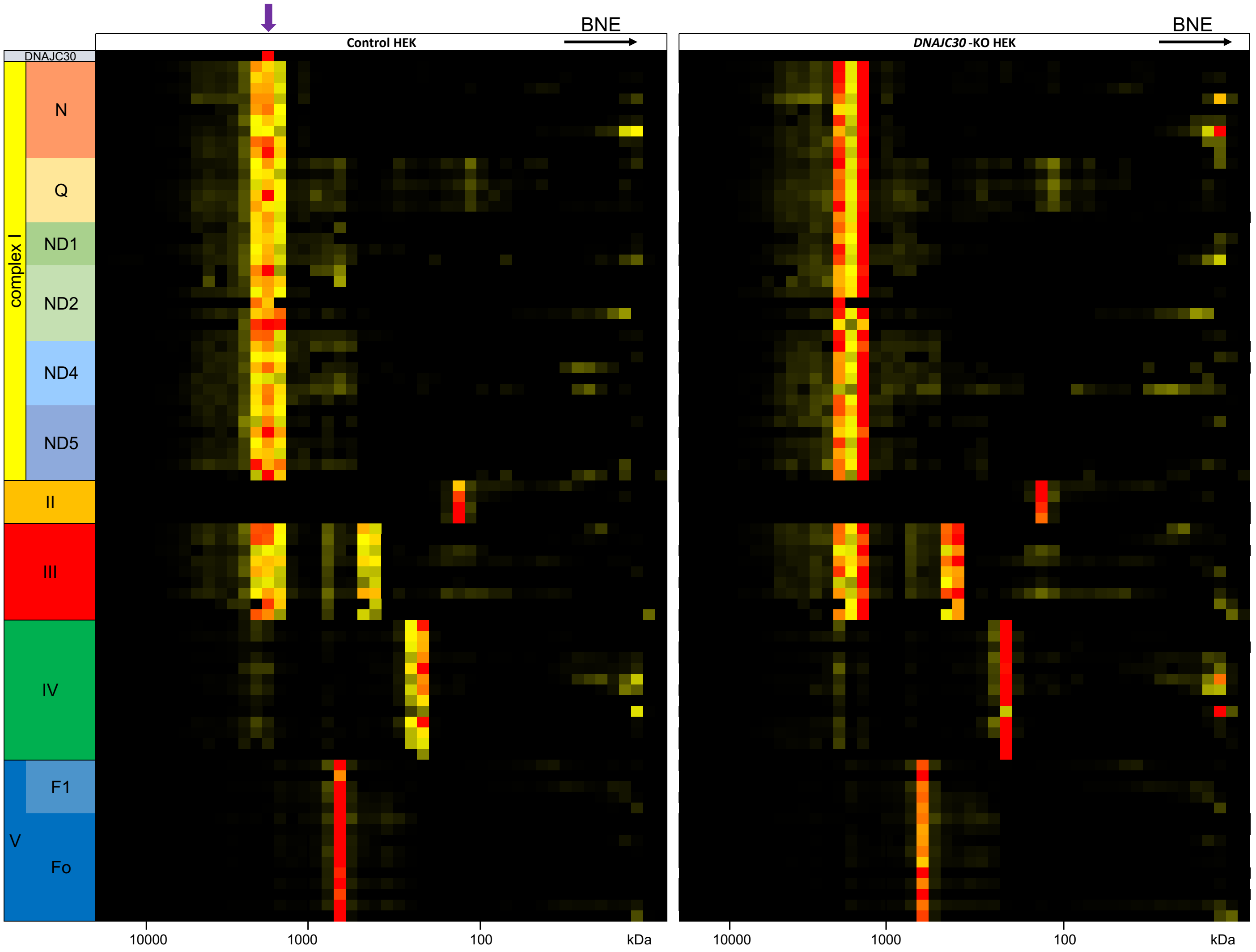

Control HEK
BNE
DNAJC30-KO HEK
BNE
DNAJC30
complex I
N
Q
ND1
ND2
ND4
ND5
II
III
IV
V
F1
Fo
10000
1000
100
kDa

F

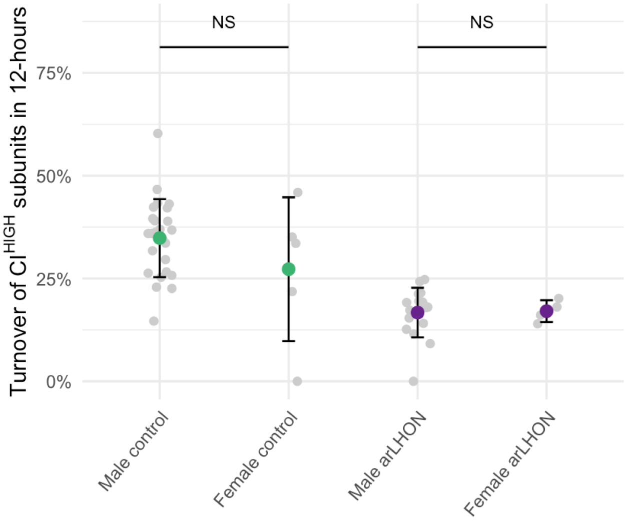

Turnover of CI^{HIGH} subunits in 12-hours
NS
NS
75%
50%
25%
0%
Male control
Female control
Male arLHON
Female arLHON

G

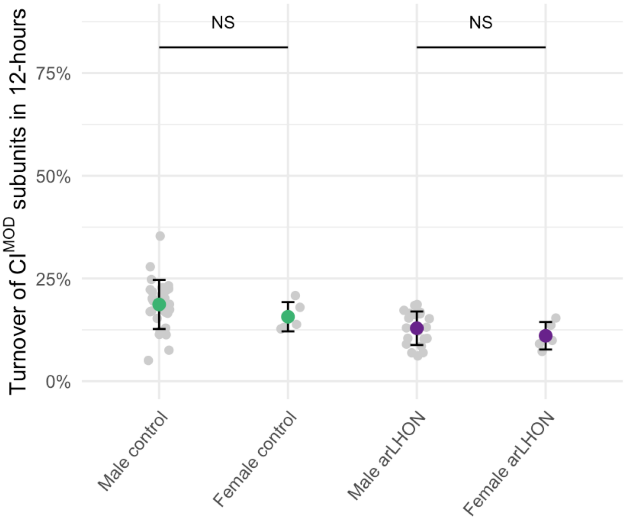

Turnover of CI^{MOD} subunits in 12-hours
NS
NS
75%
50%
25%
0%
Male control
Female control
Male arLHON
Female arLHON

H

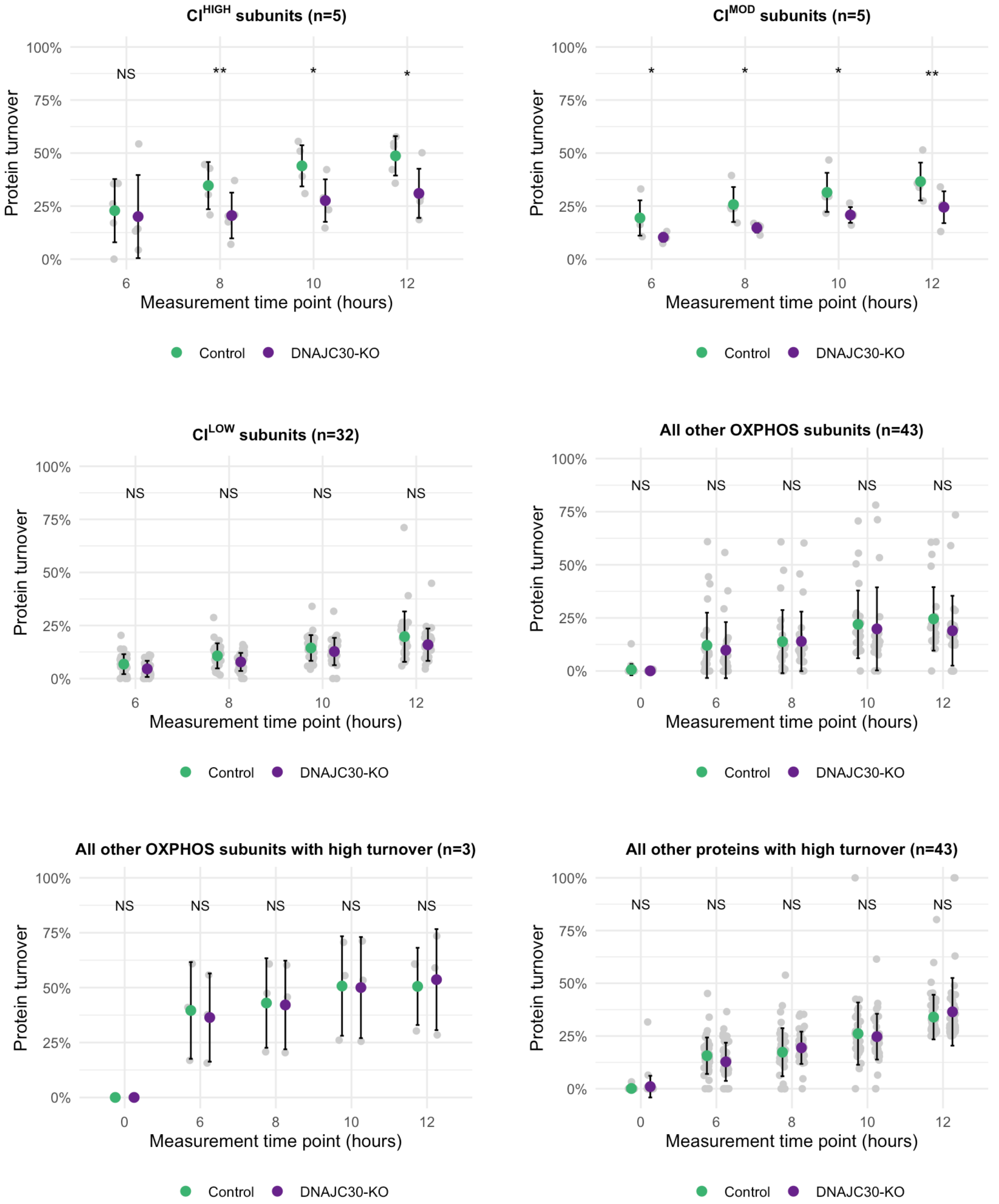

CI^HIGH subunits (n=5)
CI^MOD subunits (n=5)
CI^LOW subunits (n=32)
All other OXPHOS subunits (n=43)
All other OXPHOS subunits with high turnover (n=3)
All other proteins with high turnover (n=43)
Protein turnover
100%
75%
50%
25%
0%
NS
**
*
0
6
8
10
12
Measurement time point (hours)
Control
DNAJC30-KO

Supplementary Figure 3. *DNAJC30* mutations result in increased abundance of mitochondrial complex I and impaired repair of specific mitochondrial complex I subunits.

(A) Enriched mitochondrial pathways (as per MitoPathways3.0) in patient-derived (n=3) in comparison to control (n=105) fibroblast cell lines in the TMT-labelled quantitative proteomics data (n=5562 proteins detected in all samples) depicting the normalized enrichment score (NES) for all pathways with an adjusted $p<0.05$. The NES is depicted in purple when the core enrichment included CI subunits. **(B)** Relative specific activity of mitochondrial CI in fibroblast cell lines (control n=189, *DNAJC30* mutations n=71, technical replicates) demonstrates reduced relative specific activity of CI in association with *DNAJC30* mutations (mean 0.68, s.d. 0.11, $p<0.0001$). Data are depicted by the mean ± s.d.; two-sided Student's t-test. This analysis demonstrates the accumulation of lowly functioning CI in association with *DNAJC30* mutations. **(C)** Quantitative proteomic analysis of assembled RCC separated by blue native electrophoresis (BNE) in the control and *DNAJC30*-KO HEK cell lines solubilized in DDM (detergent dodecyl maltoside), confirming a specific CI accumulation in association with knock-out of *DNAJC30* (31 CI subunits detected, p 0.0002, nine CIII subunits detected, p 0.38, nine CIV subunits detected, p 0.50). The abundance of CI subunits (iBAQ) is depicted with the mean ± s.d.; two-sided Student's t-test. These proteomics data are available in ProteomeXchange via the PRIDE (2) partner repository with the identifier PXD022339. **(D)** Complexome profiling of fibroblast cell lines from an adult male control (left panel) and a patient with *DNAJC30* mutations (Subject 3-1, p.Tyr51Cys, middle panel) reveals normal assembly of the respiratory chain complexes in DNAJC30 defect. DNAJC30 in the control cell line runs with the CI containing supercomplex (marked by the purple arrow) indicating an interaction (left panel, top row, grey). DNAJC30 is absent in the patient cell line in-keeping with the result of the TMT-labelled quantitative proteomics for the p.Tyr51Cys variant (see **Figure S1F**). In contrast, an CI assembly factor defect (ACAD9 defect, right panel) demonstrates a severe decrease in assembled CI with accumulation of assembly intermediates, where bound assembly factors in the Q module intermediate (Q_{int}) are seen at 300 kDa (yellow box) in addition to a shift of CIII and CIV (red box) from the supercomplex to their individual complexes and small supercomplexes including CIII and IV. Such accumulation at 300 kDa was not identified in DNAJC30 defect (lower right panel). **(E)** Complexome profiling of the control and *DNAJC30*-KO HEK cell line reveals normal assembly of the respiratory chain complexes in DNAJC30 defect. DNAJC30 in the control cell line runs with the CI containing supercomplex (marked by the purple arrow) indicating an interaction. **(F)** Turnover measurement of CI^{HIGH} subunits (n=5) and **(G)** CI^{MOD} subunits (n=5) by 12-hours in control (n=7, 6 male, 1 female) and arLHON (n=6, 5 male, 1 female) fibroblast cell lines. The defect shows no predilection to gender to account for the difference in gender dependent penetrance male CI^{HIGH} subunits (mean 34.8%. ± 9.5% s.d., female CI^{HIGH} subunits, mean 27.3%. ± 17.5% s.d.). Data depicted as the mean ± s.d.; two-sided Student's t-test. A complete summary of the data is provided in **Table S10** and the experiment is depicted in **Table S11-S15** and **S17**. **(H)** Individual protein turnover (%) at 6, 8, 10, and 12-hours in control and *DNAJC30*-KO HEK cell lines across CI^{HIGH} subunits, CI^{MOD} subunits, CI^{LOW} subunits, all other OXPHOS subunits including those demonstrated to have high turnover (defined as >25% by 12-hours in the control fibroblast cell lines), and all other captured proteins demonstrated to have with high turnover. This analysis demonstrates the defect in protein turnover associated with defects in *DNAJC30* to be specific to CI subunits, specifically the CI^{HIGH} and CI^{MOD} subunits.

Supplemental methods

Sanger sequencing of *DNAJC30*. The entire gene was sequenced in two overlapping segments. The primer sequences used for PCR were: CAACCGACTCCTCTCATTGG and GTGGGTCCGAGAGGTGG (first segment) and GCCTACGTGGTGCTGGG and CAAGGGTTCAGAGGCAGG (second segment).

RNA sequencing data analysis. Differential expression analysis was performed using the R Bioconductor package DESeq2 (1). For gene set enrichment analysis (GSEA) we used the "GSEA" function in the R Bioconductor package clusterProfiler (2). The Benjamini Hochberg method was applied to correct the p values for multiple testing. The MitoPathways3.0 dataset, available at https://www.broadinstitute.org/mitocarta/mitocarta30-inventory-mammalian-mitochondrial-proteins-and-pathways, was used as input to the GSEA (MitoCarta3.0 genes annotated into a hierarchy of 149 biological pathways). The RNA sequencing count data are available in **Table S7**.

TMT-labelled quantitative proteomics. Quantitative proteomics was performed at the BayBioMS core facility at the Technical University Munich, Freising, Germany. Fibroblast cell pellets containing 0.5 million cells were lysed under denaturing conditions in urea containing buffer and quantified using BCA Protein Assay Kit (Thermo Scientific). 15 µg of protein extract were further reduced, alkylated and the tryptic digest was performed using Trypsin Gold (Promega). Digests were acidified, desalted and TMT-labeling was performed according to (3) using TMT 10-plex labelling reagent (Thermo Fisher Scientific). Each TMT-batch consisted of 8 patient samples and 2 reference samples which allowed for data normalization between batches. Each TMT 10-plex peptide mix was fractionated using

trimodal mixed-mode chromatography as described by (4). LC-MS measurements were conducted on a Fusion Lumos Tribrid mass spectrometer (Thermo Fisher Scientific) which was operated in data-dependent acquisition mode and multi-notch MS3 mode. Peptide identification was performed using MaxQuant version 1.6.3.4 (5) and protein groups obtained. Data were normalized by sample-wise normalization to normalize the effect of different sample loadings across channels in a TMT 10-plex and protein-wise normalization to enable comparisons of samples across-TMT 10-plex experiments.

TMT-labelled quantitative proteomic data analysis. Differential expression analysis was performed using the R Bioconductor package limma (6). For gene set enrichment analysis (GSEA) we used the "GSEA" function in the R Bioconductor package clusterProfiler (2). The Benjamini Hochberg method was applied to correct the p values for multiple testing. The MitoPathways3.0 dataset, available at https://www.broadinstitute.org/mitocarta/mitocarta30-inventory-mammalian-mitochondrial-proteins-and-pathways, was used as input to the GSEA (MitoCarta3.0 genes annotated into a hierarchy of 149 biological pathways). The TMT-labeled quantitative proteomics data are to be made available by Kopajtich et al., (in preparation).

Relative specific activity of CI calculation: The normalized maximal respiration rate from the Respiratory Chain Assay was divided by the mean fold-change of CI subunits measured by TMT-labelled quantitative proteomics.

DNAJC30 stoichiometry from complexome profiling calculation. DNAJC30 was identified at position of supercomplexes in substoichiometric amounts. The average of IBAQ values from CI subunits was used to calculate DNAJC30 stoichiometry on supercomplexes. In the adult control we estimated around 1 DNAJC30 protein in 200 CI molecules consistent with the idea

that the chaperone binds transiently and supports protein turnover. Stoichiometry at position of supercomplex (1618 kDa). IBAQ values used for this calculation are presented in **Table S5**.

Differential turnover of protein complexes calculation. Proteins quantified in both the control and *DNAJC30* KO-HEK cell line at the 12-hour time point (n=1223) were mapped onto individual protein complexes in the CORUM (v3.0) database (7) using the coreComplexes.txt file downloaded from the CORUM database (http://mips.helmholtz-muenchen.de/corum/#download). The mitochondrial CI N-module (n=10) and CI^{HIGH} subunits identified in this study (n=5) were manually added to the dataset. The number of quantified subunits in each CORUM complex was counted, and protein complexes with <3 quantified subunits were removed. To identify specific protein complexes with differential turnover, the turnover values of all proteins in a CORUM complex were compared between the control and *DNAJC30*-KO HEK by a two-sided Student's t-test. The Benjamini Hochberg method was applied to correct the p values for multiple testing. The mean delta turnover for each complex was calculated by the mean of the delta turnover values of all identified proteins in the complex. The delta turnover values of individual proteins were calculated as: ((sum of heavy intensity-based absolute quantification (IBAQ) / sum of light and heavy IBAQ across all bands cut from the Blue Native gel in the control cell line)) - ((sum of heavy intensity-based absolute quantification (IBAQ) / sum of light and heavy IBAQ across all bands cut from the Blue Native gel in the *DNAJC30*-KO HEK cell line)).

Collection of patient case reports. Detailed clinical case reports were collected for each subject, as below.

Family 1

Female (subject 1) born as the second child of healthy unrelated parents. One healthy brother is four years older. No family history of neurological or ophthalmological disease. Uneventful pregnancy, spontaneous uncomplicated delivery. Normal development during the first four years of life, no relevant medical history.

At four years of age started to fall easily and show spastic movements of the right hand. She manifested progressive decrease of walking distance and was unable to stand up on her own from lying or sitting position. A brain MRI at age four years and three months showed bilateral T2-hyperintensities of the putamina and pedunculi cerebelli suggestive of Leigh syndrome. CSF and plasma lactate were not elevated. A slow progression of the symptoms with increasing dysarthria and loss of gait was noted. She had normal cognitive development. At age seven years muscle biopsy (fresh muscle) revealed complex I deficiency with normal morphology and normal histochemistry. Repeat brain MRI confirmed bilateral necrosis of the putamen and lesions in the pedunculi cerebelli without progression from the MRI at four years of age. MR spectroscopy revealed a moderate lactate peak in the lesions. Myelinization was normal for her age and there were no imaging abnormalities of the optic nerve. The patient was treated with coenzyme Q10 (10 mg/kg/day), L-carnitine 50 mg/kg/day, riboflavin 5 mg/kg/day, and baclofen 2 x 30 mg/day. She had a fat-rich diet with around 60% of caloric intake from lipids. Since the age 18 years she suffers from Crohn's enterocolitis. Several cardiological, ophthalmological and ENT examinations were normal. During the following years she developed severe spasticity mainly of the extremities and lost most of her motor capacities. At the age 21 years she is unable to walk and is wheelchair-dependent. She communicates with the aid of an eye-controlled computer. Her cognitive development seems adequate.

mtDNA depletion and deletion screening was negative. Screening of the complete mtDNA was negative.

Family 2

At 19 years of age this male patient (subject 2) from Luxembourg experienced a subacute onset of blurred vision in the central visual field OD and 12 weeks later OS. Visual loss without pain or apparent difficulties in color perception progressed in both eyes with a time to nadir of ten months and eight months, in OD and OS, respectively.

The family history is unremarkable for ophthalmological or neurological diseases. Both parents, his brother, and two sisters are clinically healthy. There was a history of cigarette smoking of four pack-years at the time of disease onset, but no alcohol consumption. Past medical history revealed recurrent headache attacks since the age of ten years and a post-infectious polyneuritis cranialis and brainstem encephalitis at the age of ten years clinically presenting with dizziness, diplopia, nystagmus, nausea, and headaches. Brain MRI at that time showed unspecific signal hyperintensity of the pons. There was full remission of symptoms under treatment with high-dosage intravenous (i.v.) immunoglobulins.

Ophthalmologic examinations around eight weeks after first clinical symptoms revealed a central scotoma OD and a visual acuity of logMAR 1.30 OD and 0.00 OS. Papillary prominence was present in OD > OS and brain MRI showed a dilated optic nerve sheath diameter OS. VEP showed bilaterally increased P100 latencies. Further neurological

examinations were unremarkable including lumbar puncture to exclude intracranial hypertension.

Repeated ophthalmological and neurological examinations at around six weeks after first symptom onset in the second eye (OS) showed papillary prominence (optic disc swelling) and hyperemia (OS>OD), capillary ectasia and peripapillary vessel tortuosity (OS>OD) without capillary leakage on fluorescein angiography, temporal optic disc pallor of OD, a bilateral central scotoma and, over two weeks, a progressive dilation of optic nerve sheath diameters in ocular ultrasonography (OS>OD). VEP were not detectable anymore. Visual acuity was logMAR 1.30 in both eyes. Brain MRI performed eight weeks after first symptom onset of the second eye (OS) showed bilateral signal hyperintensities of the proximal optic nerve, the optic chiasma and the optic tract (T2-, FLAIR sequences), no contrast enhancement, no increased optic nerve sheath diameters and no swelling of the optic nerves.

At ten months after disease onset there was a mild bilateral nasal papillary prominence and a mild bitemporal optic atrophy. Visual fields showed a progressive central scotoma in both eyes (OD>OS). OCT demonstrated a bilateral, predominantly temporal decrease of retinal nerve fiber layer thickness (RNFL). Visual acuity was logMAR 1.60 OD and 1.40 OS.

At 15 months after disease onset ophthalmological examinations showed a decline of the bilateral nasal optic disc swelling and a more pronounced bitemporal optic atrophy. OCT demonstrated a progressive bilateral RNFL atrophy. Bilateral central scotoma was stable. Visual acuity was logMAR 1.40 OD and 1.22 OS.

At 5.5 years after disease onset ophthalmological examinations still showed a very mild nasal papillary prominence and a further increase of bitemporal optic nerve pallor. OCT demonstrated an associated progressive bilateral RNFL atrophy with temporal predominance. Visual acuity was off-chart with logMAR 1.68 in both eyes.

For treatment, the patient received i.v. high-dosage steroids, i.v. high-dosage immunoglobulins and immunoadsorption after first onset of symptoms OD without any improvement. Idebenone was started four weeks after clinical onset of the second eye at a dosage of 900 mg/day (Named-Patient Program, *Santhera*). The treatment was stopped after 12 months due to a lack of clinically significant response.

Suspected for LHON but negative for the common LHON causal mtDNA mutations, the patient underwent lumbar puncture and skeletal muscle biopsy eight weeks after clinical affection of the second eye. Muscle biopsy showed a prominent granular staining in oxidative enzyme reactions suspicious of mitochondrial proliferation and/or mitochondrial enlargement. Immunohistochemistry demonstrated slightly reduced staining of complex I subunits compared to controls. Biochemical examinations showed an isolated complex I defect in skeletal muscle homogenate, however no signs of mitochondrial proliferation due to normal citrate synthetase activities.

Complete sequence analysis of mtDNA and screening for mtDNA deletions by long-range PCR in muscle tissue were negative. mtDNA copy number determination in skeletal muscle tissue was normal. Whole-exome sequencing was performed.

Family 3

Subject 3-1 was first seen in clinic at 30 years of age. He is the eldest of two brothers in a family from Poland, originally from Ternopil (former Poland, currently Ukraine), with suspected consanguinity as the families of the mother and of the father came from the same area and consanguinity is not certain but deemed as likely by the family.

Clinical onset was at 29 years of age, with a defect in the central visual field (OS), at first small, which then progressed and within two weeks significantly disturbed vision. No trigger could be identified. This progressed further with worsening of the visual loss, blurred vision in the central visual field and altered color vision, exclusively OS in the first two months, interfering with normal daily life after two weeks, and continuing to progress for the next three months.

Perimetry showed central scotoma bilaterally, Pattern VEP bilaterally not reproducible, Flash VEP showed increased latencies bilaterally. Angiography fluorescein and multifocal ERG were performed. MRI of the brain and orbits and CSF studies were unremarkable. He was initially prescribed anti-inflammatory eye drops, vitamin B complex, vitamin B6 and vitamin B12, and did not improve. A tentative diagnosis of optic neuritis was made and i.v. high-dose corticoids followed by oral corticoids given, with no effect. From March 2015 on, the symptoms also involved the right eye, with a rapid progression in the next two weeks. From then on, there was a stability of the symptoms. A second trial of i.v. high-dose corticoids followed by oral corticoids led to no change. A treatment with i.v. bolus followed by oral cyclosporine did not help and had to be stopped due to increased liver function tests. Given the similar pathology presented by his brother one decade before, a diagnosis of Leber's hereditary optic neuropathy was proposed and treatment with idebenone 900 mg/day was taken five

months after clinical onset, for a 22-month period, when it was stopped due to lack of significant clinical improvement.

Currently he uses visual aids with voice-over and needs a cane when walking in unknown streets. The vision in the visual field periphery is subjectively much less affected. Colors are perceived as less intense and he cannot distinguish between hues, for example red from pink, or yellow from orange. Red, green and blue have always been recognizable, although paler than before. He also describes light sensitivity. Previously he was a full-time cabinet-maker, stopped due to the visual disability and currently works in a pottery for four hours daily.

Relevant medical history included hypercholesterolemia and folic acid deficiency. He had increased liver function tests a few months following onset, due to cyclosporine, which resolved after stopping this medication. The patient smoked 20 cigarettes per day from 19 to 22 years of age, afterwards only sporadically (3 pack-years), and stopped after onset of symptoms. He drinks about 1-2 beers (0.5 l) per week and never used recreational drugs.

General examination was unremarkable. Neurological examination showed bilateral visual impairment, impaired smooth pursuit bilaterally, central scotoma on confrontation, and bilateral temporal optic atrophy, otherwise it was unremarkable.

The genetic examination excluded the three more frequent pathogenic mutations of the mitochondrial DNA (m.11778G>A, m.3460G>A, m.14484T>C) and the complete mtDNA sequencing showed no pathogenic mutation but the presence of haplotype J. In both subjects, exome-sequencing and a skin biopsy were performed.

Subject 3-2 is the younger brother of subject 3-1, first seen in clinic at 30 years old. He had symptom onset at 17 years, of progressive bilateral decrease of visual acuity, described as blurriness in the center of vision, with worsening in the first 3.5 months then stability, with accompanying color vision problems and no other symptoms. Perimetry showed central scotoma bilaterally, pattern VEP showed OD increased P100 latency, OS no reproducible result. An MRI brain and orbits with contrast, MRI spine, CSF studies, serologies, immune screening, and antibodies to AQP4 were all unremarkable. A tentative diagnosis of retrobulbar optic neuritis was made and a course of i.v. high-dose corticosteroid therapy had no effect. He had Trental i.v. and a course of doxycycline. The diagnosis of Leber's hereditary optic neuropathy (LHON) was proposed. The patient was never treated with idebenone. At nadir the visual acuity was logMAR 2.00 (counting fingers) bilaterally, he could only see shadows and contours, everything was grey and blurry, markedly in the center of vision, less so in the periphery of vision. After three months of stability, a very slow improvement of vision was noticed and documented in the following 6 months, very significant in daily life. In the past eleven years there has been no change, both subjectively and in VA (visual acuity) tests. Currently he can read a newspaper without visual aids, he can watch TV, he can recognize bus numbers, he can recognize someone at 15 meters distance, he still needs approximation to read small print, has more difficulty reading with some contrasts between background and printed letter, has difficulty distinguishing certain colors only during formal color examination, and has no light sensitivity. He does not use visual aids other than his prescription glasses. He has a 6-month-old healthy daughter. Both parents are healthy. Other than his older brother, there are no other cases of visual impairment in the family. He never smoked and drinks about 5 beers (0.5 l) per week and never used drugs.

General examination was unremarkable, other than vitiligo in the hands. Neurological examination showed bilateral visual loss (logMAR 0.50 OD and 0.30 OS), smooth pursuit was impaired bilaterally, central scotoma on confrontation.

Genetic examination showed none of the three more frequent pathogenic mutations of the mitochondrial DNA. At 30 years of age, exome sequencing and a skin biopsy were performed.

Family 4

At 16 years of age this male patient (subject 4) experienced sudden onset of blurred vision in the central visual field of the left eye (OS) and one week later in the right eye (OD), without pain or apparent difficulties in color perception. He and his family are from Romania, with a family history unremarkable for ophthalmic or neurological diseases. His sister has type 1 diabetes. He did not smoke cigarettes nor drink alcohol.

Initial ophthalmologic examinations, one month after clinical onset, revealed visual acuity of logMAR 1.30 in both eyes and fundus examination was described as normal. Visual fields showed a central scotoma in both eyes, larger OS. Neurological examination was unremarkable for other focal deficits. Brain and orbit MRI and brain and muscle MR-spectroscopy were unremarkable.

Blood examinations including C-reactive protein (CRP), vitamin B12 and folate dosage, serological markers of autoimmunity, anti-aquaporin-4 (AQP4) and anti-MOG antibodies search were negative. Infectious etiologies were excluded.

The visual impairment of the patient continuously progressed during the first year. The OCT performed, one year after onset showed general atrophy of retinal nerve fiber layer (RNFL) in both eyes and visual acuity was off-chart with logMAR 1.68. He received i.v. corticosteroids without improvement. Idebenone was started two months after clinical onset at dosage of 900 mg/day.

At our last evaluation, two years after onset, visual acuity was logMAR 1.10 in both eyes and visual fields showed a slight improvement bilaterally.

Suspected for Leber's hereditary optic neuropathy (LHON) but negative for the common mtDNA LHON causal mutations the patient underwent complete sequence analysis of mitochondrial DNA (mtDNA) and screening by NGS targeted panel for the main genes associated with optic neuropathies, which were both negative. Whole-exome sequencing was performed.

Family 5

At 20 years of age this male patient (subject 5) experienced sudden onset of blurred vision in the central visual field of both eyes, worse in the OD, without pain. He has Russian origins, but as he was adopted, his family history is not available. He reported sporadic use of cannabis and cocaine, and abuse of alcohol. He smoked 10 cigarettes/day.

Serum C-reactive protein (CRP) and anti-MOG antibodies search were negative. Oligoclonal bands were negative in CSF and serum.

Initial ophthalmologic examinations revealed visual acuity of logMAR 2.00 (counting fingers) at 30 cm in both eyes and bilateral pseudoedema of the optic disc at fundus examination. The OCT performed 3 months after onset revealed severe temporal atrophy of the RNFL in both eyes with relative sparing of the fibers in the other quadrants.

The patient received oral corticosteroids without improvement. Suspected for LHON but negative for the common mtDNA mutations, the patient underwent a targeted investigation on the DNAJC30 gene, considering that he was an adopted child in Italy but his geographical origin was from Russia. This analysis revealed the genetic defect common to all other patients of our case series. Idebenone was started (900 mg/day) in 6 months following onset, after the genetic diagnosis. At our last evaluation visual acuity was logMAR 1.30 OD and 0.50 OS without any further thinning of RNLF in the temporal quadrants except for a slight decrease in the superior quadrants at the OCT examination.

Family 6

At 14 years of age this male patient (subject 6) experienced sudden onset of blurred vision on the right eye followed one month later by the left eye. Visual acuity at first evaluation, two months after onset, was logMAR 2.00 (counting fingers) OD and 1.00 OS. He has Russian origins, but because he was adopted his family history is not available. He did not smoke cigarettes.

Serum C-reactive protein (CRP), serum vitamin B12 and folate, and serological markers of autoimmunity were negative. Brain and orbit MRI were normal.

The patient received i.v. steroids and i.v. and immunoglobulin therapy (30 g/day for 4 days) without improvement. Nine months after onset the patient noticed vision improvement. Idebenone was started (540 mg/day) one year after clinical onset. At last evaluation there was a complete recovery of visual acuity in the left eye (logMAR 0.00) and partial improvement in the right eye (logMAR 1.00).

Suspected for LHON, but negative for the common LHON causal mtDNA mutations, the patient underwent complete sequence analysis of mtDNA and screening by NGS-targeted panel for the main genes associated with optic neuropathies, which were both negative. Whole-exome sequencing was performed.

Family 7

At 17 years of age this male patient (subject 7) noticed sudden onset of blurred vision on the right eye. He was born after uncomplicated pregnancy and delivery from unrelated parents, who come from a small village with 3500 inhabitants near Kiev in the Ukraine. He does not smoke cigarettes or drink alcohol. He was initially treated with steroids and i.v. immunoglobulin without clinical benefit. About a month later the second eye (OS) became affected.

Ophthalmologic examination revealed bilateral temporal pallor and bilateral papilledema at fundus oculi examination and central scotoma in both eyes at computerized visual field. Two months after clinical onset, therapy with idebenone (945 mg/day) was started. Visual impairment worsened in the following months, and last visual acuity was logMAR 2.30 (hand

movement) in both eyes, five months after onset. OCT revealed bilateral optic atrophy with a reduction of the RNFL thickness (55 um OS and 56 um OD).

Suspected for LHON but negative for the common LHON causal mtDNA mutations, the patient underwent complete sequence analysis of mtDNA and screening by NGS-targeted panel for the main genes associated with optic neuropathies, which were both negative.

Family 8

At 38 years of age this patient (subject 8) came to the clinic reporting sudden painless bilateral visual loss four month before the visit. There were no specific findings in previous medical history. The patient smokes and reports mild to moderate alcohol consumption. Ophthalmologic examination revealed severe visual impairment (logMAR 1.00 bilaterally), central scotomas of both eyes, dyschromatopsia, and GCC (ganglion cell complex) thinning. The patient started idebenone treatment (90 mg per day). Three month later he came for a follow-up visit and presented with significant vision recovery (logMAR 0.30 bilaterally). At the next follow-up visit three more month later he had further recovery of visual acuity to logMAR 0.1 bilaterally (80% of normal visual acuity rate).

Family 9

The patient (subject 9) first experienced visual impairment at the age of 13 when he presented with sudden bilateral painless visual loss. Three month later he came to the Institute of Eye Diseases with severe visual impairment: logMAR 1.40 OD and 1.50 OS and central scotomas of both eyes. Starting from the next follow-up visit he showed slight progressive vision

recovery up to logMAR 0.40 OD and 0.00 OS. During the last visit, 11 years after the first visit, he had logMAR 0.54 OD and 0.00 OS (28% and 100% of normal visual acuity rate). The patient did not receive idebenone treatment.

Family 10

At the age of 13 this patient (subject 10) experienced onset symptoms of visual impairment in the left eye followed two month later by the right eye. There were no specific findings in previous medical history or in family history. The first ophthalmological examination performed found impaired visual acuity logMAR 1.1 bilaterally, optic nerve disc pallor and central scotomas. OCT revealed a loss of GCC and RNFL thickness. Starting from the next visit he began to show slight vision recovery. One year after the first visit the VA became stable and remains so to today (logMAR 0.00 bilaterally, 100% of normal visual acuity rate). The patient received idebenone treatment (90 mg per day).

Family 11

This patient (subject 11) first came to the Institute of Eye Diseases at the age of 24 and reported sudden painless bilateral loss of vision. During the examination poor visual acuity was found (logMAR 2.0 bilaterally). Optic coherent tomography showed an increased thickness of the peripapillar retinal nerve fibers layer in the temporal quadrant and thinning of the macular ganglion cell complex, which are characteristic for the early stage of LHON. To date, the patient has had no improvement in visual acuity. At the last visit, six years after onset, his VA was logMAR 1.92 OD and 1.51 OS (1 % and 3% of normal visual acuity rate). He took idebenone 900 mg per day during the first 5 years of the disease.

Family 12

This patient (subject 12) had onset of symptoms at the age of 15 years, when he first experienced bilateral visual loss. At the first visit, one month after onset, his VA was logMAR 1.40 bilaterally and color perception test revealed severe dyschromatopsia. He started idebenone treatment (90 mg per day). Since then his VA and color perception began to improve slightly. 10 months later, VA was logMAR 0.48 OD and 0.70 OS. Ophthalmologic examination during the last visit, 6.5 years after onset, revealed VA logMAR 0.30 OD and 0.90 OS (50% and 1% of normal visual acuity rate).

Family 13

At the age of 19 the patient (subject 13) presented with blurred vision in the right eye. 16 weeks later the left eye became involved. First ophthalmological examination revealed VA logMAR 1.40 bilaterally. 10 month later he came for the next follow-up visit having a significant VA improvement to logMAR 0.70 OD and 0.90 OS. He started idebenone treatment (90 mg per day). At the last follow-up visit nine years after the onset his VA was stable (logMAR 0.70 OD and 0.70 OS) although he reported to have not taken idebenone on a regular basis (20% of normal visual acuity rate).

Family 14

At the age of 28 this patient (subject 14) presented to the clinic reporting bilateral painless visual loss two weeks before the visit. MRI did not reveal any pathological findings. The patient reported to have been a moderate smoker but stopped several months before onset. He also

reported daily alcohol consumption before the onset of visual loss. Ophthalmological examination revealed impaired VA logMAR 1.00 OD and 0.48 OS, central scotomas, severe impairment of color perception and thinning of GCC predominantly in the right eye. RNFL thickness stayed higher than the normal range for a significant time and only 10 months after the first visit decrease of RNFL was noticed which was more significant in the temporal part of the disc. The patients had an enlarged optic nerve disc, and therefore thick RNFL. The RNFL remained for a longtime within normal findings on OCT during the next several follow-up visits. Four months after onset he came to see the doctor reporting decline of visual acuity (logMAR 1.40 bilaterally). At the last visit, 11 months after onset, the VA was logMAR 0.00 OD and 0.10 OS (100 % and 80% of normal visual acuity rate). He continues to take idebenone treatment (90 mg per day).

Family 15

The patient (subject 15-1) first experienced visual impairment at the age of 17 year when he presented with sudden bilateral painless visual loss. He came to the clinic with visual acuity logMAR 0.30 OD and 0.10 OS, central scotomas of both eyes, and severe impairment of color perception. Two month later, during the next follow-up visit, severe visual acuity impairment was found logMAR 1.90 OD and 1.20 OS. Four months later he showed a slight VA improvement to logMAR 1.11 bilaterally. The patient did not return for a follow-up appointment until 11 years after onset where his visual acuity was logMAR 0.18 bilaterally (66 % of normal visual acuity rate).

The patient’s sister (subject 15-2) was also referred to the clinic at the age of 25 years with symptoms consistent with a diagnosis of LHON (visual acuity logMAR 1.51 OD and 1.40 OS).

We could not however perform genetic analysis nor further clinical examination. The patient reportedly experienced recovery of visual acuity, as reported by her mother. Given the phenotype of the patient and the genetic finding in the sibling, this patient is assumed to carry the same homozygous variant in *DNAJC30*.

Family 16

This female patient (subject 16) first experienced the symptoms consistent with LHON at the age of 40 years, when she presented with low visual acuity (off-chart logMAR 1.68 bilaterally). At the next visit six month later, she had visual acuities of logMAR 1.50 bilaterally. At the last visit, nine years after onset, she showed slight visual acuity improvement in the right eye to logMAR 1.30 and slight deterioration in the left eye to logMAR 1.60 (5% and 2.5% of normal visual acuity rate). The patient reported irregular compliance with prescribed treatment and no idebenone intake.

Family 17

At 15 years of age this patient (subject 17) came to the clinic reporting bilateral painless visual loss. MRI revealed no pathological findings. The patient suffered from hemolytic-uremic syndrome (HUS) and underwent kidney allotransplantation. Since transplantation he has been taking immunosuppression therapy. Ophthalmological examination revealed visual acuities of logMAR 1.10 bilaterally and optic nerve disc pallor. Four month later, he had visual acuities of logMAR 1.20 OD and 1.00 OS. He started idebenone treatment (90 mg per day). Since this time, his visual acuity remains more or less stable. At the last visit, 14 years after onset, the

visual acuity was logMAR 1.31 OD and 1.11 OS (5% and 8% of normal visual acuity rate). During the last visit the patient reported irregular compliance with prescribed treatment.

Family 18

Subject 18-2 is a 26-year-old male and the maternal nephew of subject 18-1. At age 16 years he presented to an emergency department for sudden and marked decrease in visual acuity. Visual acuity was off-chart with logMAR 1.68 OD and 1.30 OS with a central scotoma bilaterally. Visual evoked potentials were reported to be severely altered. Marked temporal optic nerve pallor with otherwise normal retinal aspect at the fundus was recorded. T2-hyperintensities were seen on brain MRI. He received i.v. corticosteroids. A partial yet significant clinical recovery was recorded in the following three weeks, with VA of logMAR 0.30 bilaterally. At last follow-up at 25 years his VA was logMAR 0.10 bilaterally. Mitochondrial DNA screening yielded negative results and so whole exome sequencing was performed.

Subject 18-1 is a 53-year-old male born in Tunisia. He moved to France around the age of 35. Medical history revealed an episode of VA decrease around the age of 6-10 years and spontaneous recovery. At 41 years of age he was referred for decreased visual acuity, difficulties in distance vision and gradual constriction of the visual field said to have started 2-3 years ago. VA measured to logMAR 0.10 bilaterally. 4.5 years later, at 45.5 years, VA of the OD was unchanged but VA of the OS had dropped to logMAR 0.40.

Optic atrophy at the fundus, bilateral tubular visual field, and red-green dyschromatopsia. Neurological workup was unremarkable, with the exception of low pupillary reflex and

ophthalmoplegia, with inability to move the eyes up and laterally. 17 months later, at 46.9 years, he consulted for collapse of VA to logMAR 1.00 bilaterally. He complained of recurrent headaches treated by paracetamol and ibuprofen. Marked and almost generalized bilateral loss of optic nerve fibers at the RNFL on OCT were recorded. Normal flash ERG but altered checkerboard pattern ERG with absent N95 wave attesting marked optic nerve dysfunction further supported by abnormal and delayed PEV responses. One year later, at 48.2 years, VA reduced to logMAR 1.30 OD and 2.00 (counting fingers) OS, with tubular visual field. Dramatic and generalized loss of optic nerve fibers at the RNFL OCT.

Family 19

Subject 19 is 32-year-old male. A rapid decrease of VA was reported over one month at the age of 15 years. His VA was logMAR 0.60 OD and 1.30 OS. Goldmann perimetry was normal, as was the ERG.

Ophthalmoscopy revealed bilateral optic atrophy and peripapillary telangiectasias. Metabolic workup was unremarkable. Six months later his OD VA dropped to 0.70, whereas OS the VA remained unchanged. Goldmann perimetry revealed bilateral large centrocecal scotoma. ERG was normal. A blood sample was collected and the patient was lost unfortunately to follow-up.

Family 20

At the age of 19 years this otherwise healthy male patient (subject 20-1) experienced an acute onset of painless bilateral vision impairment. Initially, the symptoms manifested as bilaterally discretely blurred vision alongside with difficulties in color perception and reduced sensitivity

to contrast. The symptoms progressed into severe bilateral vision loss within 12 months. No additional symptoms were noted. No trigger could be identified. The patient denied cigarette or alcohol consumption.

Ophthalmological examination at the time of nadir revealed a bilateral and severe visual impairment (visual acuity logMAR 1.00/1.00). VEPs were absent. Funduscopic examination identified (only) a discrete pallor of the temporal sections of the optical nerves bilaterally and was otherwise normal. CSF analysis detected oligoclonal bands. Brain imaging via MRI was unremarkable, especially without indications of inflammatory lesions within the brain matter or signs of inflammation of the optic nerves. A treatment with i.v. steroids (1g prednisolone/ 5 days) failed to improve symptoms.

A clinical suspicion of LHON was followed up by testing of the common causal LHON mutations and subsequent sequence analysis of the entire mtDNA which both revealed unremarkable results.

The previous medical history was reported uneventful except for a short episode with blurred vision around the age of 8 years. At that time no ophthalmological examination was conducted and the symptoms resolved spontaneously after a duration of approximately 3-4 months.

Our patient is the youngest of four children (2 male, 2 female) of healthy consanguineous parents from Turkey. A severe and intermittent visual impairment of the patient’s brother (subject 20-2) who is currently 31 years of age, was stated, which was described to have started at 12 years of age and showed spontaneous recovery to 80% of the previous visual acuity after

a period of 1-2 years. The family history was otherwise unremarkable for ophthalmological or neurological disease conditions.

Family 21

The patient (subject 21), currently 21 years of age, attended the clinic in September 2019 with a history of visual loss starting in March 2019 for OD and in June 2019 for OS. At the time of examination his VA was OD logMAR 0.98 and OS logMAR 1.26. He could not read 16 and 22 colour tables of 27 (OD and OS, respectively). Central scotomas and thinning of the complex of ganglion cells (CGC) and RNFL were detected. Brain MRI showed no pathology. He received 90-180 mg/day of Idebenone. Repeated examination in January 2020 revealed improvement in visual acuity, especially on OS VA (OD logMAR 0.87, OS logMAR 0.28) and increase in light sensitivity according to static perimeter data. However, the dyschromatopsy remained the same and the structural changes in the form of GCC and RNFL thinning had increased.

Family 22

The patient (subject 22), currently 22 years of age, attended the clinic in July 2019 due to visual loss 4 months prior for OD, and 10 days later for OS. VA OD logMAR 1.04 and OS logMAR1.00. The examination revealed central scotomas of OU, dyschromatopsy (OU could not read 12 tables out of 27), thinning of retinal CGC and RNFL for both eyes. Brain MRI showed no pathology. He received 90-180 mg/day of Idebenone. When examining the patient again after 4 months, their visual acuity and colour vision improved: VA OD logMAR 0.59,

OS logMAR 0.41. OD he could not read 5 tables out of 27, OS he could read all the 27 tables. Structural changes in the form of GCC and RNFL thinning had increased.

Family 23

The patient (subject 23) came to the clinic in October 2019 with complaints of decreased vision in June 2019 for OS, then for OD. At the time of examination, VA OD logMAR 1.09, OS logMAR1.35, and he could not read 22 and 26 tables out of 27 (for OD and OS, respectively), central scotomas and thinning of the complex of ganglion cells (CGC) and RNFL were detected. Brain MRI showed no pathology. He received 90-180 mg/day of idebenone. A repeated examination in December 2019 revealed a slight improvement in the visual acuity (VA OD logMAR 0.98, OS logMAR 1.03), and colour vision (could not read 20 and 21.5 tables out of 27, respectively, for OD and OS), central scotomas remained. Structural changes in the form of GCC and RNFL thinning had increased.

Family 24

The patient (subject 24), currently 16 years of age, came to the clinic in October 2019 complaining of a spot in his vision OD, which had appeared 5 months before. The examination revealed central scotomas more pronounced in OD, mild dyschromatopsy in OD (couldn't read 2 tables out of 27), thinning of the retinal ganglion cell complex (GCC) in both eyes, but VA OU logMAR 0.00. 6 months after the onset, the patient complained about reduced vision in OS and measured VA was OD logMAR 0.27, OS logMAR 0.02. There was a negative trend in perimeter indexes for both eyes, as well as increased loss of CGC and RNFL. Slight

dyschromatopsy is still detected only for OD (could not read 4.5 out of 27 tables), no change of colour vision detected for OS. Next visit scheduled in 1 month.

Family 25

The patient (subject 25), currently 16 years of age, came to the clinic in May 2005 complaining of a a sharp simultaneous vision loss in April 2005. In June 2005, VA OU logMAR 2.0 (counting fingers). Considering the ophthalmoscopic findings that are typical for LHON, the patient was recommended for genetic examination of three frequent and nine rare mutations of mtDNA. No mutations were detected. In 2011, in repeated examination, VA OU logMAR 0. MRI scan of the brain revealed no pathology. However, from the ophthalmoscopic findings looks like optic nerve atrophy, the patient cannot read 3 out of 27 tables, 1 out of 27 for OS. A small central scotoma and thinning of the RNFL was detected. Now we now no longer in contact with the patient.

Family 26

The patient (subject 26) complained in 2012 of having had reduced vision since 2002. They linked their low vision to frequent alcohol consumption. VA OU logMAR 1.0. Central scotomas, pronounced dyschromatopsy, thinning of RNFL were detected. Considering the ophthalmoscopic findings that are typical for LHON, the patient was recommended a genetic examination of frequent and 9 rare mutations of mtDNA. No mutations were revealed. The patient was invited to carry out a new examination, which is to be carried out within a month.

Family 27

In 2010, the patient (subject 27) complained about OD vision decline, which he had noticed 4 years before, at 12 years of age. In 2005, he had also noticed painless vision loss in OS (up to VA OS logMAR 1.11) with subsequent recovery to VA OS logMAR 0.00. During examination, VA OD logMAR 0.10, VA OS logMAR 0.00. Mild OD dyschromatopsy was detected, the patient reads the tables slowly, OS: normal. OD: central scotoma within 5°, OS: area of vision not altered. Thinning of RNFL was noted for OU. The patient was referred to genetic examination of frequent and 9 rare mutations of mtDNA. No mutations were detected. At the repeated examination in 2012, VA OD logMAR 0.18, VA OS logMAR 0.00. There is no contact with the patient at present (phone numbers changed).

Family 28

The proband (subject 28) is a 12 years and 3 months old male, first of two children born to non-consanguineous parents of Ukrainian descent. He was born following uneventful pregnancy and delivery, and achieved developmental milestones adequately. He was reportedly asymptomatic until the age of 11 years, at which he began experiencing episodes suspected to be seizures. At 11 years and 10 months of age, the patient experienced a sudden vision loss in his OD, with a central scotoma. Similar symptoms involving the OS ensued three months later.

He underwent extensive neuro-ophthalmological investigations, which did not yield a clear etiology. Ophthalmological evaluation at nadir showed logMAR 0.4 in both eyes, visual fields (HVF 24-2 stim V) demonstrated temporal field defect OD and paracentral scotoma OS. Brain MRI showed thinning of the optic discs bilaterally, and was otherwise normal. Metabolic

workup, including serum lactate and pyruvate, ceruloplasmin, very long chain fatty acids (VLCFA) and urine organic acids profile, was within normal limits. In terms of his suspected seizures, he is not currently treated with anti-epileptic drugs.

With a working diagnosis of LHON, treatment with idebenone was initiated at 900 mg/day at 11 years and 10 months. Repeated ophthalmological evaluation (5 months after initiation of treatment) revealed improvement: OD logMAR 0.1 and OS logMAR 0.0, visual fields demonstrated improvement with minimal temporal field defect OD and small nasal field defect OS.

Genetic evaluation included Sanger sequencing of the common LHON causal mtDNA mutations, as well as two NGS targeted panels for genes associated with LHON and/or optic atrophy, all deemed negative. Finally, single whole exome sequencing was pursued for the proband.

Family 29

The proband (subject 29) is a 15 years and 3 months old male, first of two children born to non-consanguineous parents of Ukrainian descent. He was born following uneventful pregnancy and delivery, and achieved developmental milestones adequately. At 5 years and 6 months he was first examined by an ophthalmologist due to blurry vision, and began to wear glasses. At the age of 15 years he experienced sudden-onset central vision loss in his OS. While he did not complain of vision loss in the OD, evaluation including OCT at presentation did reveal bilateral central scotomas. He is cognitively intact, with no extraocular signs or symptoms.

An extensive evaluation ensued, including a brain MRI which was considered normal, as well as tests for infectious and inflammatory etiologies. CSF was tested for oligoclonal bands as well as for lactate and amino acids profile, all were within normal limits.

With the suspicion of LHON, he underwent single whole exome sequencing, including sequencing of the mtDNA. He has yet to begin idebenone treatment.

References

1. Love, M. I., Huber, W., & Anders, S. (2014). Moderated estimation of fold change and dispersion for RNA-seq data with DESeq2. Genome biology, 15(12), 550.
2. Yu, G., Wang, L. G., Han, Y., & He, Q. Y. (2012). clusterProfiler: an R package for comparing biological themes among gene clusters. Omics: a journal of integrative biology, 16(5), 284-287.
3. Zecha, J., Satpathy, S., Kanashova, T., Avanessian, S. C., Kane, M. H., Clauser, K. R., ... & Kuster, B. (2019). TMT labeling for the masses: a robust and cost-efficient, in-solution labeling approach. Molecular & Cellular Proteomics, 18(7), 1468-1478.
4. Yu, P., Petzoldt, S., Wilhelm, M., Zolg, D. P., Zheng, R., Sun, X., ... & Kuster, B. (2017). Trimodal mixed mode chromatography that enables efficient offline two-dimensional peptide fractionation for proteome analysis. Analytical chemistry, 89(17), 8884-8891.
5. Tyanova, S., Temu, T., & Cox, J. (2016). The MaxQuant computational platform for mass spectrometry-based shotgun proteomics. Nature protocols, 11(12), 2301.

6. Ritchie, M. E., Phipson, B., Wu, D. I., Hu, Y., Law, C. W., Shi, W., & Smyth, G. K. (2015). limma powers differential expression analyses for RNA-sequencing and microarray studies. Nucleic acids research, 43(7), e47-e47.
7. Giurgiu, M., Reinhard, J., Brauner, B., Dunger-Kaltenbach, I., Fobo, G., Frishman, G., ... & Ruepp, A. (2019). CORUM: the comprehensive resource of mammalian protein complexes—2019. Nucleic acids research, 47(D1), D559-D563.
